# Synergistic Rh/La Codoping Enables Trap-Mediated Charge Separation in Layered Perovskite Photocatalysts

**DOI:** 10.1021/jacs.5c12425

**Published:** 2025-10-13

**Authors:** Mengqi Duan, Shuai Guo, Wentian Niu, Hangjuan Ren, Thomas Dittrich, Dongpei Ye, Lucy Saunders, Sarah Day, Veronica Celorrio, Diego Gianolio, Peixi Cong, Robert S. Weatherup, Robert Taylor, Songhua Cai, Yiyang Li, Shik Chi Edman Tsang

**Affiliations:** † Wolfson Catalysis Centre, Department of Chemistry, 6396 University of Oxford, Oxford OX1 3QR, U.K.; ‡ Department of Applied Physics, 26680 The Hong Kong Polytechnic University, Kowloon 999077, Hong Kong SAR China; § Helmholtz-Zentrum Berlin für Materialien und Energie GmbH, Schwarzschildstr. 8, 12489 Berlin, Germany; ∥ 120796 Diamond Light Source Ltd., Harwell Science & Innovation Campus, Didcot OX11 0DE, U.K.; ⊥ Department of Materials, University of Oxford, Parks Road, Oxford OX1 3PH, U.K.; # Clarendon Laboratory, Department of Physics, University of Oxford, Oxford OX1 3PU, U.K.

## Abstract

Two-dimensional layered perovskite oxides have emerged as promising photocatalysts for solar-driven hydrogen evolution. Although doping has been widely employed to enhance photocatalytic performance, its role in modulating the electronic structure and the local chemical environment of these materials remains poorly understood. Here in this study, we investigate the codoping of Rh and La into exfoliated nanosheets of the Dion–Jacobson perovskite KCa_2_Nb_3_O_10_ to enhance photocatalytic hydrogen evolution reaction (HER) activity. A substantial increase in H_2_ evolution rate, from 12.3 to 69.0 μmol h^–1^, was achieved at an optimal doping level of 0.2 wt % Rh and 1.3 wt % La. Comprehensive structural and spectroscopic analyses, including synchrotron techniques and high-resolution microscopy, revealed that Rh^3+^ substitutes Nb^5+^ to introduce shallow 4*d* acceptor states that mediate charge separation, while La^3+^ substitutes Ca^2+^, compensates for aliovalent charge imbalance, and modulates local lattice distortions and oxygen vacancy formation. This codoping strategy enhances charge carrier lifetime and separation efficiency through a trap-mediated mechanism. The observed volcano-shaped activity trend highlights a narrow compositional window, where electronic and structural factors are optimally balanced. These findings establish a mechanistic foundation for defect engineering in layered perovskites and offer a pathway for the rational design of photocatalysts.

## Graphical abstract

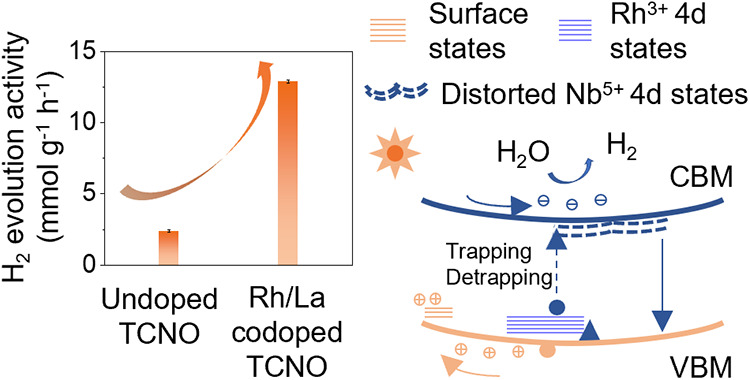

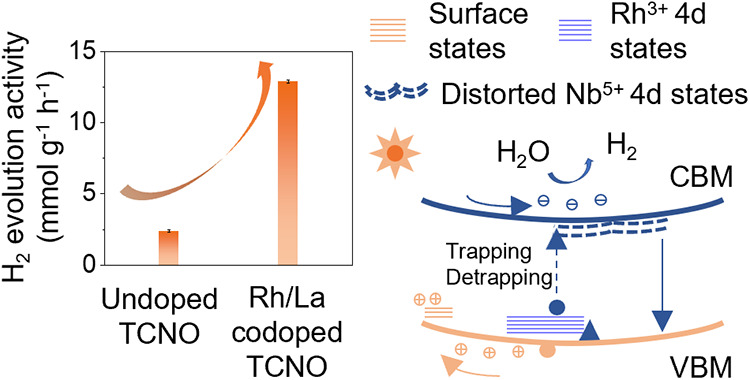

## Introduction

Photocatalytic water splitting using semiconductor metal oxides offers a promising approach to converting solar energy into green hydrogen.^1–3^ Despite extensive efforts, achieving high catalytic activity remains a major scientific challenge. This limitation arises primarily from fast photogenerated charge carrier recombination and slow surface reaction kinetics.^4^ Therefore, innovative materials and design strategies are still urgently required to overcome current limitations. Layered perovskite oxides present an attractive platform for photocatalysis due to their structural tunability and stability. These layered perovskites consist of negatively charged two-dimensional (2D) slabs of corner-sharing transition-metal-oxide octahedra (B site) interleaved with alkaline earth or rare earth cations (A-site), which are weakly connected with interlayer cations (A′ site) through electrostatic interactions.^5^ This unique structure enables exfoliation of three-dimensional (3D) layered perovskites into 2D nanosheets by substituting interlayer A′ site cations with bulky organic cations, which can improve their photocatalytic performance by leveraging their unique properties. First, the surface area can be dramatically increased, and more active sites would be exposed after exfoliation, improving catalytic reaction kinetics at the surface. Besides, the quantum confinement effect in 2D nanosheets can shift the conduction and valence bands, thereby increasing the redox driving force. It also restricts carrier motion perpendicular to the sheet, enhancing charge separation and the charge carrier lifetime. Furthermore, the migration distance of the photogenerated charge carriers from bulk to surface would be largely shortened, which would reduce charge carrier recombination.^6^ Additionally, the flexibility of isomorphous substituting A and B site metal cations endows layered perovskites with tunable physicochemical properties. Among various exfoliated 2D layered perovskite oxide nanosheets, tetrabutylammonium (TBA) intercalated TBACa_2_Nb_3_O_10_ nanosheets (TCNO) derived from KCa_2_Nb_3_O_10_ (KCNO) layered perovskite shows outstanding photocatalytic activity toward hydrogen evolution reaction (HER) when compared with other unmodified 2D perovskite oxide nanosheets, likely due to its band position thermodynamically favorable for HER and internal electric field induced by tilted NbO_6_ octahedra beneficial for charge separation.^7^ However, its photocatalytic activity remains limited due to poor solar light absorption caused by its large optical bandgap energy (i.e., 3.6–3.8 eV) and fast charge carrier recombination.^8,9^

A wide range of strategies have been studied to extend the absorption range of solar light and facilitate the charge separation in photocatalysts, including doping,^10^ constructing heterostructures,^11^ loading cocatalysts,^12^ introducing functional groups on the surface,^13^ and building up an external electric/magnetic field.^14^ Among these, doping, even in small amounts, has been particularly effective in tuning photocatalytic activity by altering electronic and structural properties of catalysts, thanks to the differences in electronic configuration, size, or electronegativity between the dopant and host atoms.^15,16^ For example, Okamoto et al. found that Rh doping significantly enhanced the photocatalytic activity of TCNO nanosheets for HER, and they attributed this improvement to the electron capture effect of Rh^3+^, which inhibited photogenerated charge carrier recombination.^17^ Codoping strategies have further advanced this concept by introducing complementary dopants that together modulate defect formation and band structure, which have been extensively studied in binary metal oxide and conventional perovskite (ABO_3_) photocatalysts. For example, Moss et al. revealed that in Rh and La codoped SrTiO_3_, La^3+^ suppressed the formation of deep states associated with Rh^4+^ and reorganized the electronic structure, which extended the photogenerated charge carrier lifetime and enhanced the photocatalytic activity.^18^ However, investigations about codoping strategies on layered perovskite are scarce. One notable example is the work by Ohmagari et al., who demonstrated that Rh–La codoping was more effective than other combinations in enhancing the photocatalytic HER activity of TCNO.^19^ Nevertheless, the exact roles of dopants and their synergistic effects remain poorly understood, largely due to the intrinsic complexity of these systems. Their impact is often broadly attributed to changes in light absorption, charge separation and transport, or surface reactivity without a detailed mechanistic explanation (Supporting Table 1). The intricate interplay between structural and electronic factors introduced by doping still needs to be unraveled. Therefore, there is a pressing need for deeper insights into the underlying reaction mechanisms.^20^

In this study, Rh and La were codoped into TCNO nanosheets to enhance photocatalytic HER activity and the underlying mechanism was systematically investigated. Rh and La were chosen for their ability to substitute host cations while maintaining structural stability, as indicated by a favorable Goldschmidt tolerance factor (*t* = 0.94). La^3+^ (1.36 Å, CN = 12) closely matches Ca^2+^ (1.34 Å) at the A-site, and Rh^3+^ (0.67 Å, CN = 6) is comparable to Nb^5+^ (0.64 Å) at the B-site. In addition, Rh^3+^ introduces shallow acceptor levels due to the energy alignment between its 4d orbitals and O^2–^ 2p states, facilitating photocatalysis.^17^ In contrast, La^3+^ serves as a charge compensator, enhancing Rh^3+^ dispersion without directly contributing electronic states.^21^ A combination of advanced structural and electronic characterization techniques, such as synchrotron X-ray diffraction (SXRD) and high-angle annular dark-field scanning transmission electron microscopy (HAADF-STEM), confirmed that Rh^3+^ substitutes Nb^5+^ at the B-site, introducing shallow acceptor levels within the bandgap, while La^3+^ substitutes Ca^2+^ at the A-site, modulating local electronic structure by suppressing oxygen vacancies and distorting NbO_6_ octahedra. Furthermore, time-resolved photoluminescence (TRPL) spectroscopy and modulated surface photovoltage spectroscopy (SPV) revealed a synergistic effect of Rh and La codoping on enhancing charge separation and prolonging the charge carrier lifetime via Rh^3+^ 4d states. As a result, TCNO nanosheets achieved a 5-fold increase in photocatalytic HER, achieving a high H_2_ evolution rate of 69.0 μmol h^–1^ at the optimal Rh/La codoping level. This understanding reveals the intricate interplay between electronic structure and photogenerated charge carrier dynamics modulated by codopants in 2D layered perovskite nanosheets, offering new insights into the rational design of defect-engineered photocatalysts.

## Results and Discussion

A top-down method was used to synthesize TCNO nanosheets with different Rh and La doping concentrations (Figure 1). XRD and scanning electron microscopy (SEM) confirmed the successful synthesis of KCa_2_Nb_3_O_10_ (KCNO) and HCa_2_Nb_3_O_10_ (HCNO) with a rectangular cuboid morphology (Supporting Figures 1–2). Subsequent exfoliation in tetrabutylammonium hydroxide (TBAOH) resulted in the disappearance of (00*l*) (*l* > 1) diffraction peaks and the preservation of in-plane diffraction peaks such as (110), (200), and (220) (Figure 2a and Supporting Figure 3). This selective loss of periodicity along the *c*-axis, coupled with the preservation of in-plane order, confirmed successful exfoliation. In-plane diffraction peaks in SXRD patterns showed a clear and progressive shift to lower angles with increasing Rh and La contents, indicating that the substitution of host cations by Rh and La ions caused a lattice expansion (Figure 2b). Based on dopant weight percentages determined by X-ray fluorescence spectroscopy (XRF), the perovskite nanosheets were labeled as TCNO, TCNO:0.2Rh, TCNO:0.2Rh/1.3La, TCNO:0.6Rh/2.2La, and TCNO:1.8Rh/6.4La, respectively (Supporting Tables 2–3). Although the nominal doping amounts of Rh are the same for TCNO:0.2Rh, TCNO:0.2Rh/1.3La, and TCNO:0.6Rh/2.2La, the actual Rh content increased with increasing nominal La doping, suggesting that La facilitated Rh doping into the host. Scanning electron microscopy (SEM) analysis revealed the formation of thin nanosheet layers with disordered aggregation upon drying (Supporting Figures 4–5). Nitrogen adsorption–desorption isotherms confirmed a significant increase in BET surface area after exfoliation (Supporting Figures 7 and 8, Table 4). HAADF-STEM further confirmed the thin-layer morphology of the exfoliated products, as illustrated by TCNO:0.2Rh/1.3La (Figure 2c). The measured d-spacing (4.0 Å) resembled that of the (100) plane (3.9 Å) (Figure 2d). A highly dispersed substitution of La ions at the Ca sites and Rh ions at the Nb sites in TCNO nanosheets was observed (Figure 2d). Atomic-resolution HAADF-STEM images along the [001] zone axis (Figure 2f–h) revealed higher contrast for doped La and Rh atoms compared to Ca at the A-sites and Nb at the B-sites, consistent with the *Z*-contrast of HAADF-STEM images. The substitution of La and Rh into the crystal lattice was further confirmed by 3D atomic modeling and atomic contrast color-coded top-view mapping (Figure 2g,h). Energy-dispersive X-ray spectroscopy (EDX) mapping showed a homogeneous distribution of Ca, Nb, O, Rh, and La within the entire nanosheet (Figure 2i and Supporting Figure 6). Atomic force microscopy (AFM) (Supporting Figure 9) showed that the majority of nanosheets are around 3–5 nm thick, corresponding to single or double layers.^17^

**Figure 1:**
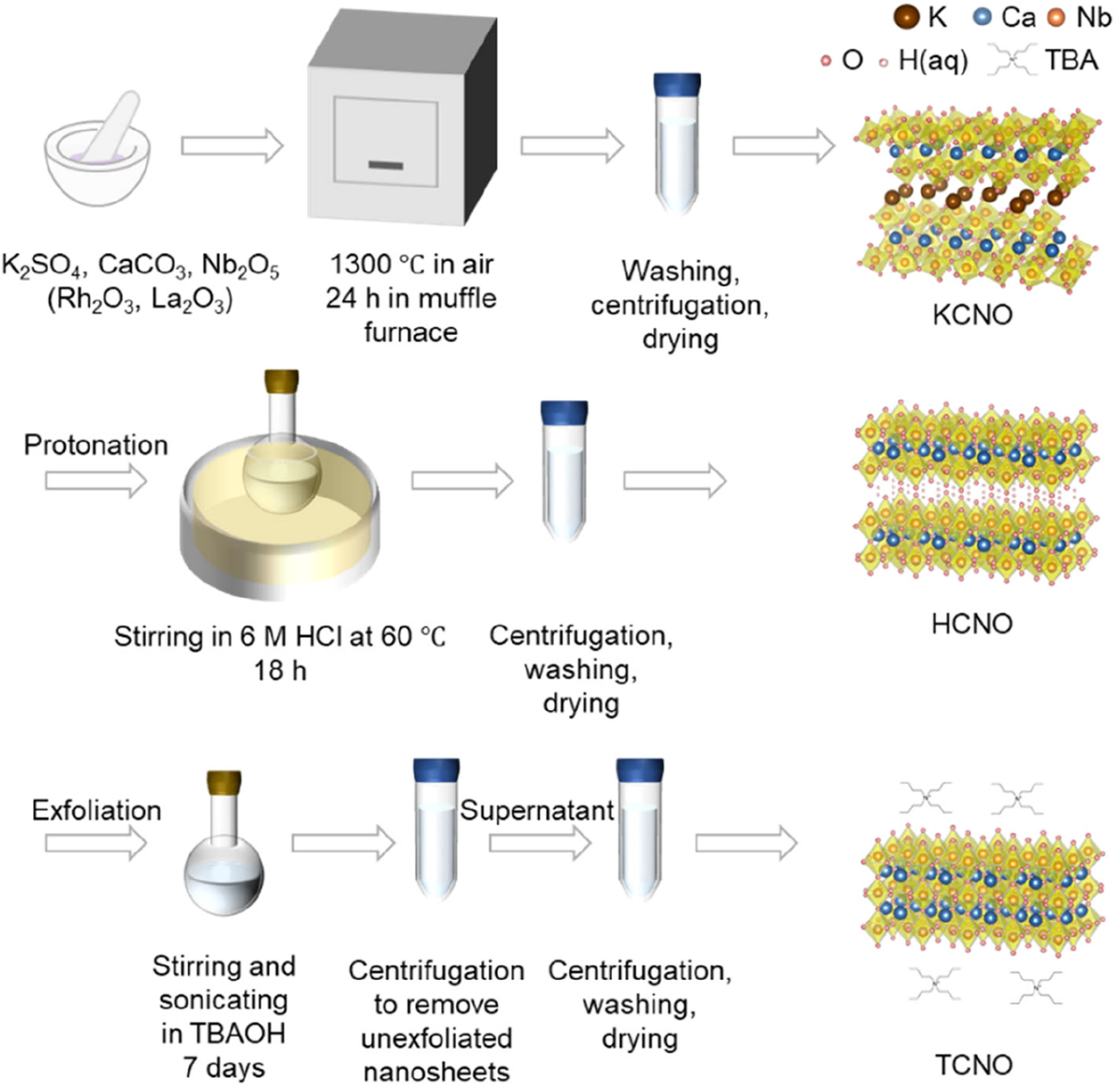
Schematic illustration of the synthesis procedure for undoped and Rh/La-doped TCNO nanosheets.

**Figure 2:**
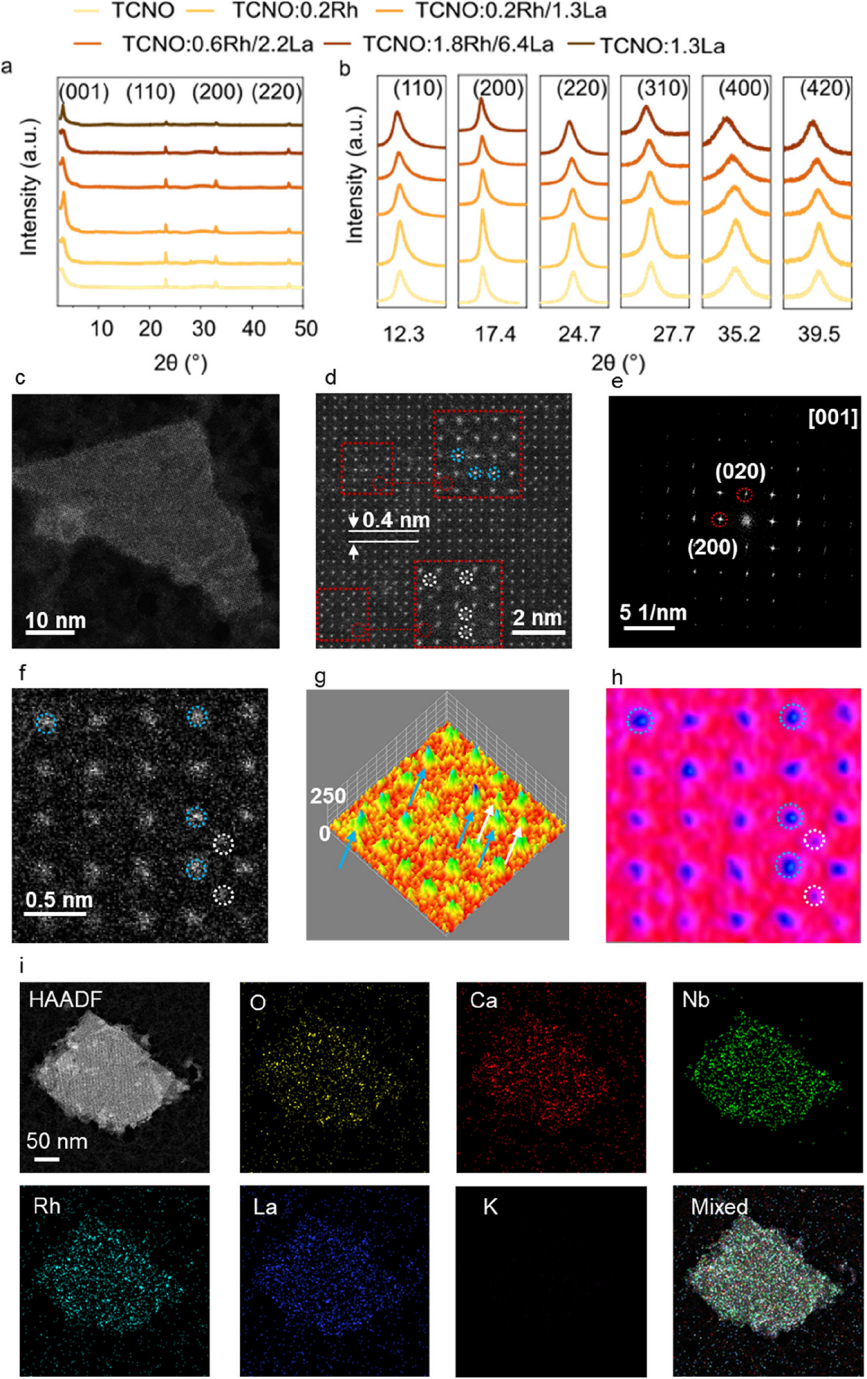
Phase identification and morphology of the exfoliated layered perovskite. (a) XRD patterns of undoped and doped TCNO. (b) SXRD patterns of undoped and doped TCNO collected using MAC detector. (c) HAADF-STEM image of TCNO:0.2Rh/1.3La. (d) High-resolution HAADF-STEM image of TCNO:0.2Rh/1.3La. Rh substituted at B site (white circles) and La substituted at A-site (blue circles) in TCNO:0.2Rh/1.3La. (e) FFT pattern through [001] zone axis of TCNO:0.2Rh/1.3La. (f) Atomic-resolution HAADF-STEM image of TCNO:0.2Rh/1.3La and the corresponding ImageJ-processed representations: (g) a 3D atomic model for height determination and (h) an atomic contrast color-coded top view for atomic position mapping. The substituted Rh and La atoms are marked by white and blue arrows and circles, respectively. (i) EDX-STEM mapping of O, Ca, Nb, Rh, La, and K in TCNO:0.2Rh/1.3La.

The photocatalytic HER activity of the nanosheets was evaluated in a closed batch reactor, revealing a volcano-shaped trend as a function of Rh and La content (Figure 3a, Supporting Figure 10). Undoped TCNO exhibited a modest H_2_ evolution rate of 12.3 μmol h^–1^. Doping with 0.2 wt % Rh (TCNO:0.2Rh) significantly boosted the activity to 62.7 μmol h^–1^, while codoping with 0.2 wt % Rh and 1.3 wt % La (TCNO:0.2Rh/1.3La) further increased the rate to 69.0 μmol h^–1^, placing it among the top-performing photocatalysts reported to date (Supporting Table 5). However, further increases in Rh and La concentrations resulted in a sharp decline in HER activity, highlighting a narrow compositional window for optimal performance. Additionally, the wavelength-dependent apparent quantum efficiency (AQE) measurements showed that the AQE decreased progressively with increasing wavelength, consistent with the absorption edge of the material, confirming that the hydrogen evolution activity is directly driven by the intrinsic photoexcitation of the Rh/La codoped TCNO nanosheets (Supporting Figure 11, Table 6, Note 3). Furthermore, application of a 420 nm long-pass filter during illumination resulted in negligible hydrogen evolution (Supporting Table 7), confirming that the activity predominantly originates from the UV-excited charge carriers. Time profile measurements over a 6 h period showed stable HER activity for both TCNO:0.2Rh and TCNO:0.2Rh/1.3La, with no observable degradation (Figure 3b,c). Interestingly, TCNO:0.2Rh/1.3La exhibited a gradual increase in activity during the first 4 h, the origin of which may need further investigation. Ten consecutive 5 h cyclic tests (totaling 50 h) were further carried out on TCNO:0.2Rh/1.3La (Figure 3d), during which the hydrogen evolution rate remained essentially unchanged, confirming the excellent catalytic durability of the material. Postreaction characterization confirmed the structural stability of these materials. In-plane XRD peaks, ultraviolet–visible (UV–vis) spectra, and HAADF-STEM images of TCNO:0.2Rh/1.3La remained identical (Supporting Figures 12–16), indicating that the perovskite framework and dopant distribution were preserved. A shift of the (001) diffraction peak to higher angles, along with a reduction in Brunauer–Emmett–Teller (BET) surface area (Supporting Figure 17), suggested partial protonation of the nanosheets during photocatalysis. TRPL spectra revealed that the lifetime of photogenerated charge carriers in postreaction TCNO:0.2Rh/1.3La remained unchanged, indicating stable charge carrier dynamics (Supporting Figure 18). Furthermore, UV–vis spectra and EDX-STEM confirmed the stability of Rh dopants, ruling out Rh leaching or reduction of Rh to metallic Rh nanoparticles (Supporting Figures 16 and 19).

**Figure 3:**
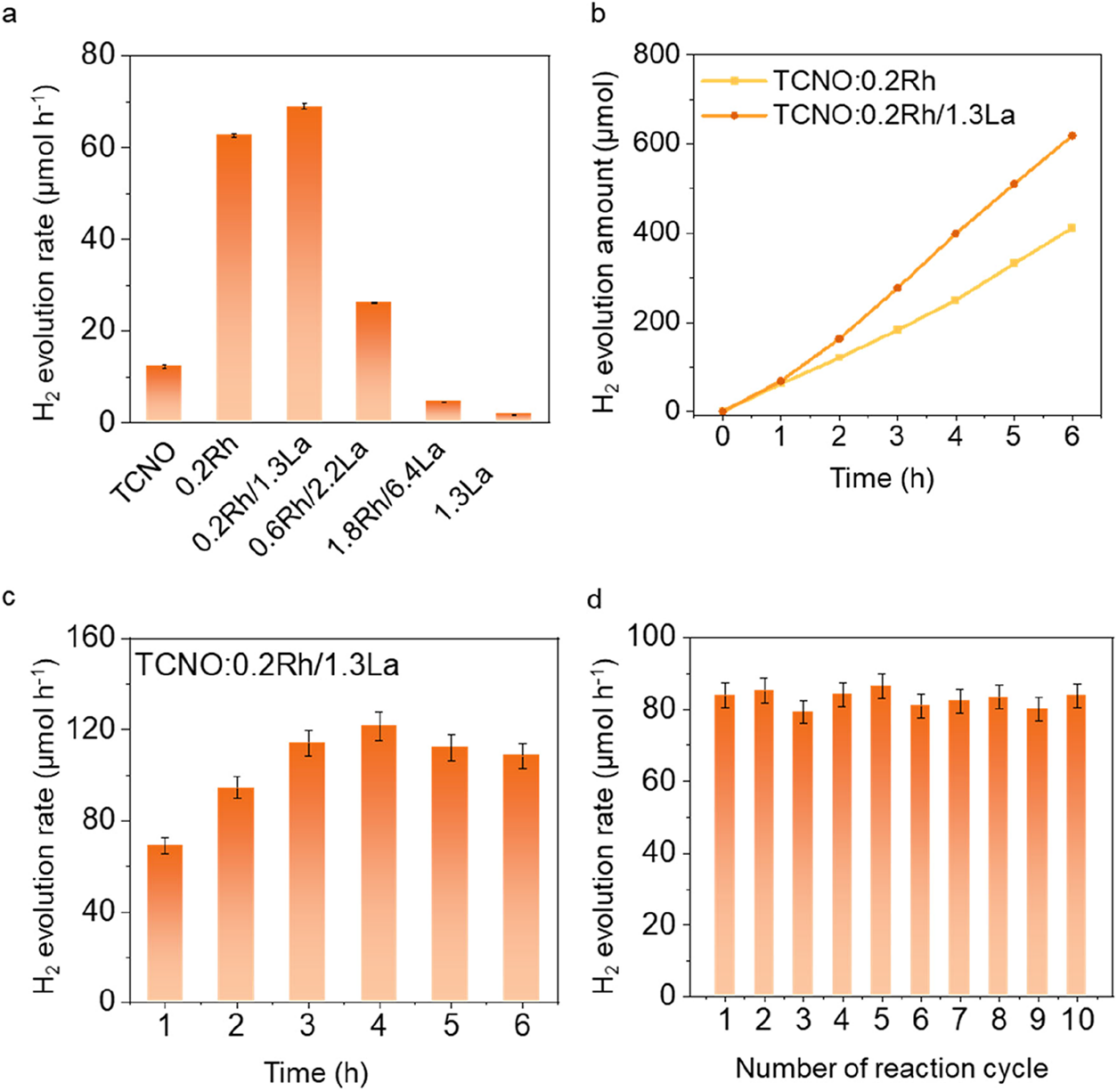
Photocatalytic performance assessment. (a) Photocatalytic H_2_ evolution rate of undoped and doped TCNO. 5 ± 0.35 mg catalyst was dispersed in 10 vol % methanol DI water solution and transferred to a batch reactor. Then, 2 bar Ar was purged into the reactor as inert gas. After 1 h illumination by a Xe lamp without any filter, the gas inside the autoclave was purged into GC three times, and the average H_2_ amount evolved during the reaction was calculated. (b) Time profile of photocatalytic activity. The H_2_ amount was measured by GC every 1 h, and the reactor was purged with Ar again after each run without taking the catalysts out of the reactor. (c) Cyclic test of TCNO:0.2Rh/1.3La. Each reaction cycle lasts 1 h. (d) Ten cyclic tests of TCNO:0.2Rh/1.3La. Each reaction cycle lasts 5 h.

The observed volcano-shaped activity trend and narrow optimal doping range underscore the complex interplay among dopants, electronic structure, and charge carrier dynamics, motivating our deeper mechanistic studies.

The bulk and surface electronic structures of Rh/La-doped samples were investigated by using X-ray absorbance spectroscopy (XAS) and X-ray photoelectron spectroscopy (XPS), respectively. The X-ray absorption near-edge structure (XANES) confirmed that the average valence state of Rh substituted at the B site is close to +3 in all Rh/La codoped TCNO as the Rh absorption edge aligned well with that in the Rh_2_O_3_ reference (Figure 4a and Supporting Figure 20).^22^ The extended X-ray absorption fine structure (EXAFS) showed a dominant first shell peak at around 1.5 Å corresponding to Rh–O bonds and no observable Rh–Rh peak at 2.4 Å, which supported the presence of isolated Rh^3+^ substitution in the bulk (Figure 4b). The corresponding EXAFS fitting showed that the coordination number of Rh is close to that of Nb (i.e., CN = 6) in TCNO:0.2Rh/1.3La (CN = 6.76 ± 0.44), TCNO:0.6Rh/2.2La (CN = 6.05 ± 0.48), and TCNO:1.8Rh/6.4La (CN = 6.05 ± 0.54), further confirming that Rh occupies the B-site (Nb site) (Supporting Figure 21 and Table 8). In addition, laboratory-based XAS measurements confirmed that the average valence state of La in the bulk is +3 (Supporting Figure 22). XPS on the corresponding HCNO confirmed that two Rh species existed, namely, Rh^0^ (307.7 eV for 3*d* 5/2) and Rh^3+^ (309.9 eV for 3*d* 5/2), on the surface of HCNO:0.2Rh, HCNO:0.2Rh/1.3La, and HCNO:0.6Rh/2.2La, and only Rh^3+^ species was detected on HCNO:1.8Rh/6.4La (Figure 4c, Supporting Figure 23, and Notes 1 and 2). Furthermore, Rh^0^ on the surface decreased while Rh^3+^ increased with increasing La content, indicating that La effectively facilitated the substitutional doping of Rh^3+^ (Supporting Tables 9–10). In XPS spectra of exfoliated layered perovskite nanosheets (Supporting Figures 24–25), only TCNO:1.8Rh/6.4La exhibited detectable Rh 3d signals, suggesting a surface-enriched distribution of Rh in the bulk layered perovskites at low La doping levels. Importantly, in TCNO:1.8Rh/6.4La, exfoliation resulted in partial reduction of surface Rh^3+^ to Rh^0^, which was considered a minor fraction of the overall Rh species due to the average oxidation state of Rh across all Rh/La codoped TCNO samples close to +3 confirmed by XANES. In addition, EPR measurements indicated that bulk KCNO and HCNO are oxygen-vacancy silent, whereas exfoliation introduces abundant oxygen vacancies (Supporting Figures 26 and 27). Notably, an increase in oxygen vacancies was observed upon Rh^3+^ doping, suggesting that the charge imbalance from aliovalent Rh^3+^ substitution is compensated by the formation of oxygen vacancies. With increasing La^3+^ doping, the concentration of oxygen vacancies decreased, becoming negligible in TCNO:0.6Rh/2.2La. However, in the heavily doped sample, TCNO:1.8Rh/6.4La, the EPR signal for oxygen vacancies intensified again, indicating that excessive doping can induce the formation of more oxygen vacancies.

**Figure 4:**
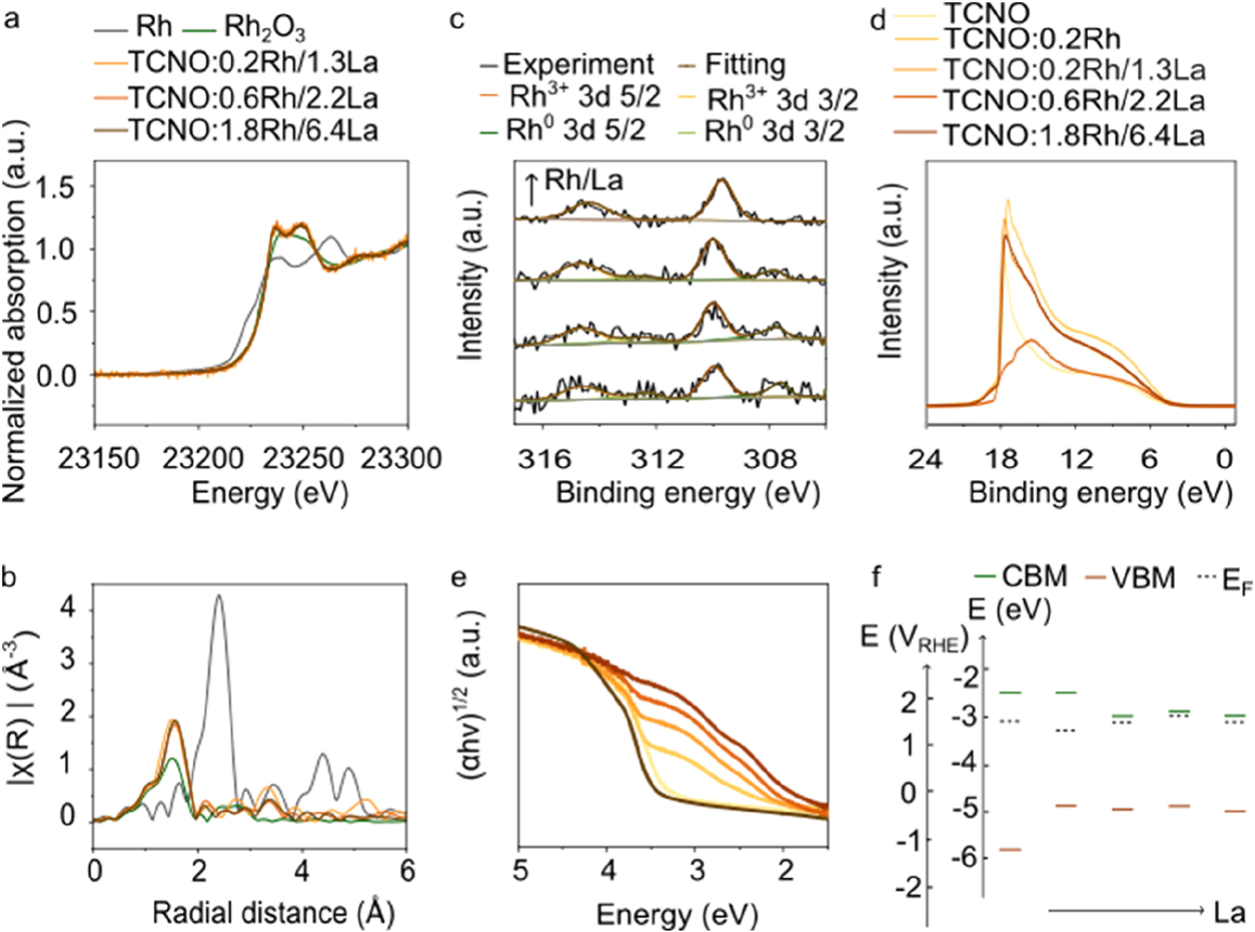
Bonding environment of Rh and electronic structures of undoped and doped calcium niobium oxides. (a) Rh *K*-edge XANES spectra of Rh- and La-doped nanosheets with reference to Rh foil and Rh_2_O_3_. (b) Fourier transform Rh *K*-edge XAFS spectra of Rh. (c) XPS narrow scan spectra of Rh 3d in undoped and doped HCNO. From bottom to top: TCNO:0.2Rh, TCNO:0.2Rh/1.3La, TCNO:0.6Rh/2.2La, and TCNO:1.8Rh/6.4La. (d) UPS spectra of undoped and doped TCNO. (e) Tauc plot converted from UV–vis DRS of undoped and doped TCNO (golden yellow curve: TCNO; orange curve: TCNO:0.2Rh; burnt orange curve: TCNO:0.2Rh/1.3La; orange-brown curve: TCNO0.6Rh/2.2La; medium brown:1.8Rh/6.4La; dark brown: TCNO:1.3La). (f) Band diagram of undoped and doped TCNO. From left to right: TCNO, TCNO:0.2Rh, TCNO:0.2Rh/1.3La, TCNO:0.6Rh/2.2La, TCNO:1.8Rh/6.4La.

The resulted electronic band structure of bulk and exfoliated layered perovskites was then analyzed using ultraviolet photoelectron spectroscopy (UPS) combined with UV–vis diffuse reflectance spectroscopy (UV–vis DRS) (Figures 4d–f and Supporting Figures 28–32).^23–25^ The measured band structure is not significantly altered upon exfoliation, which was attributed to nanosheet aggregation (as observed by SEM), mitigating the expected bandgap widening from quantum confinement. In undoped TCNO, a single absorption edge at 3.4 eV was observed, corresponding to band-to-band transitions. Upon Rh doping, a new absorption band emerged at 2.6 eV, attributed to electronic transitions from Rh^3+^ 4d states to the conduction band.^17^ Codoping with La further intensified this band, consistent with an increased level of Rh^3+^ incorporation. In addition, a new absorption edge at 2.0 eV appeared in Rh/La codoped samples, assigned to transitions involving Rh^3+^ 4d states and newly formed distorted Nb^5+^ 4d states induced by La doping.^17,26,27^ The progressive increase in the intensity of both bands with higher Rh and La contents suggested an increased density of Rh^3+^ 4d states and the formation of additional Nb^5+^ 4d states in distorted NbO_6_ octahedra. Furthermore, UV–vis DRS indicated that the Rh^3+^ 4d states are located around 0.9 eV above the valence band maximum (VBM), confirming their nature as shallow acceptor states. This was corroborated by UPS measurements, which showed a VBM shift of around 0.8–0.9 eV toward the conduction band upon Rh^3+^ doping. Although slight shifts in Fermi level (*E*_F_) and Rh^3+^ 4d states were observed in other samples with varying Rh and La content, these variations were within the instrumental energy resolution (i.e., ∼0.1–0.2 eV), indicating negligible changes in their positions (Supporting Table 11). Additionally, *E*_F_ remained much closer to the conduction band minimum (CBM) than to the VBM across all samples, confirming the n-type nature of both undoped and doped TCNO.

To further understand the influence of exfoliation and doping effect on photogenerated charge carrier dynamics, TRPL was employed to probe the average lifetime (τ_average_) (Figure 5a, Supporting Figure 33, and Table 12). TRPL revealed that exfoliation prolongs the photogenerated charge carrier lifetime, likely due to 2D confinement. TCNO:0.2Rh/1.3La showed the longest charge carrier lifetime among all of the samples. This indicates that moderate Rh/La codoping benefits charge carrier separation, but Rh-only doping and heavy Rh/La codoping lead to more serious charge carrier recombination, possibly due to increased oxygen vacancies and distorted NbO_6_, respectively, which could serve as recombination centers.^7,27,28^

**Figure 5:**
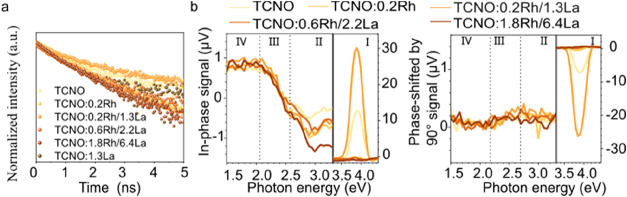
Photogenerated charge carrier dynamics. (a) Normalized TRPL spectra of undoped and doped TCNO monitored at 443 nm. (b) In-phase (left) and phase-shifted by 90° (right) SPV spectra of undoped and doped TCNO.

Complementary to TRPL, the spatial separation of photogenerated charge carriers was studied by SPV.^29–31^ Figure 5b shows the in-phase signals (X signal) (left) and phase-shifted SPV signals (Y signal) (right) of the nanosheets. Four distinct regions, denoted as region I, II, III, and IV, were identified based on changes in signal slope, each corresponding to the activation of a new electronic transition.^32^ Region I (onset around 3.5 eV) was assigned to the band transition, because the bandgap energy determined here, i.e., the onset photon energy, is consistent with the bandgap energy (3.4–3.6 eV) determined by UV–vis. Region II (onset around 2.6 eV) could be assigned to the transitions from Rh^3+^ 4d states to the conduction band, as the excitation energy matches that determined from UV–vis and UPS (around 2.7 eV). Region III (onset around 2.1 eV) could be assigned to the transitions from deep levels caused by surface states to the conduction band.^33^ Region IV (below 2.1 eV) was tentatively attributed to transitions from shallow surface states to the conduction band, yet the exact origin remains to be confirmed.

Besides, the sign of the signal indicates the separation directions of free and trapped charge carriers. In region I, the positive X signals and negative Y signals indicated that free and trapped electrons prefer moving toward the bulk, while free and trapped holes prefer moving toward the surface. This direction indicates the driving force for dominating charge separation. In the case of a well-defined n-type doped semiconductor, the driving force would be related to upward band bending in the surface space charge region. However, the SPV signals were rather low. Therefore, it seems that the driving force is mainly related to the preferential trapping of holes at surface states. In region II, negative X signal and positive Y signal suggested that free and trapped electrons originally excited from Rh^3+^ 4d states prefer to move toward the surface, whereas holes left behind at these trap states prefer moving toward the bulk. This behavior might be explained by the inherently higher mobility of excited electrons in the conduction band than trapped holes at Rh^3+^ 4d states with a slower diffusion rate. In regions III and IV, doping does not make observable changes to SPV signal, indicating that doping has little influence in this region.

Additionally, the magnitude of signals provides information on separation rates and effective densities of charge carriers. In region I, Rh doping significantly decreased the X and Y signal magnitudes, but upon moderate Rh/La codoping, both X and Y signal magnitudes significantly increased. However, further increasing Rh/La doping significantly decreased the X and Y signal magnitudes. This indicates that moderate Rh/La codoping could effectively facilitate both free and trap charge carrier separation, while Rh-only doping and excessive Rh/La codoping lead to a severe charge recombination and decrease effective charge carrier separation in space. This change is inherently coherent to the lifetime changes indicated by TRPL. In region II, the X signal magnitude increased with increasing Rh^3+^ 4d state density, and the Y signal is almost zero, indicating the trapped holes could readily detrap from Rh^3+^ 4d states and diffuse into the valence band. Combining magnitude changes in regions I and II, we hypothesized that under the cooperation of La, Rh^3+^ 4d states could effectively mediate the bandgap excited charge separation due to the fast detrapping process of holes. On one hand, La would decrease the recombination at oxygen vacancies in Rh-only doping. On the other hand, excessive La doping distorted the structures, where distorted NbO_6_ octahedra give rise to lower excited states than others, leading to a strong local potential fluctuation, facilitating recombination and resulting in the decreased SPV signals.^27^

The analysis presented above reveals a clear synergistic effect of Rh and La codoping in TCNO. Rh acts as an electronically active dopant, introducing acceptor states above the VBM, while La functions primarily as a structural modifier, regulating local lattice distortions and defect formation. In TCNO:0.2Rh (Figure 6a), the aliovalent substitution of Nb^5+^ by Rh^3+^ introduces Rh^3+^ 4d states above the VBM, along with oxygen vacancies to compensate for the charge imbalance. These Rh^3+^ 4d states facilitate trap-mediated charge separation. However, the concomitant oxygen vacancies may undermine this effect by serving as recombination centers or hindering hole transfer within the valence band. On the other hand, these vacancies can also act as active adsorption sites for reactants, thereby enhancing surface reaction kinetics. Although the charge carrier lifetime and separation efficiency are reduced, the accelerated surface kinetics may partially compensate for these drawbacks, resulting in a significant increase in the photocatalytic hydrogen evolution activity.

**Figure 6:**
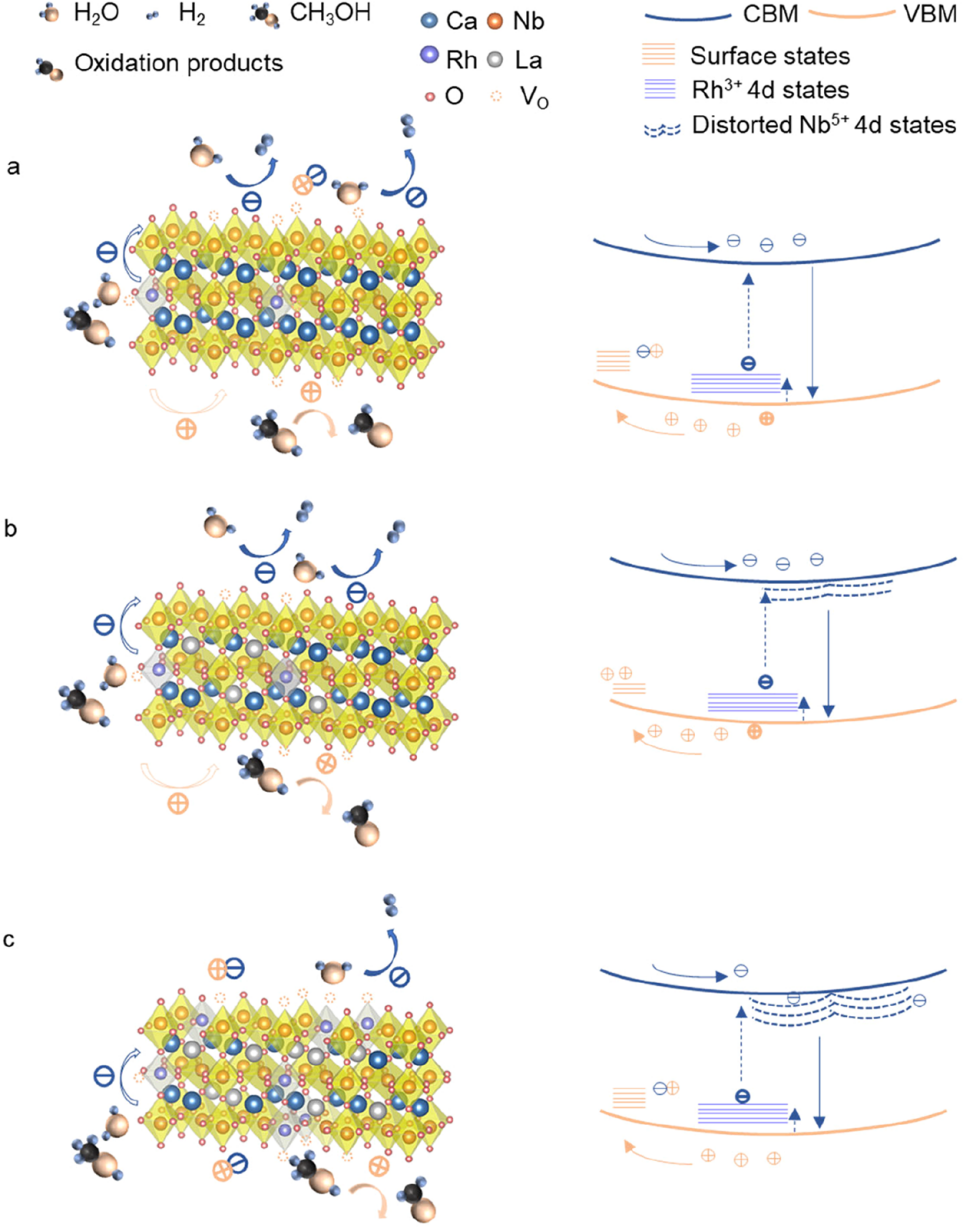
Schematic illustration of the proposed charge separation and loss mechanism in Rh/La-doped TCNO nanosheets. (a) Rh-doped TCNO–Rh^3+^ introduces shallow 4d acceptor states that mediate charge separation, but accompanying oxygen vacancies may act as recombination centers or hinder hole transport. (b) Moderately Rh/La codoped TCNO–La^3+^ compensates for charge imbalance and suppresses oxygen vacancies, enhancing charge separation and prolonging carrier lifetime via Rh^3+^ 4d trap states. (c) Heavily Rh/La codoped TCNO–Excessive La-induced lattice distortion and local potential fluctuations promote charge recombination, reducing carrier mobility and photocatalytic activity.

Introducing La^3+^ as a codopant (Figure 6b) allows charge compensation for Rh^3+^ substitution by replacing Ca^2+^, effectively suppressing oxygen vacancy formation. This enhances charge separation via Rh^3+^ 4d trap states and extends the charge carrier lifetime. However, the reduction in surface vacancies may slow the surface reaction kinetics, which explains why the photocatalytic HER activity of TCNO:0.2Rh/1.3La remains comparable to that of TCNO:0.2Rh despite improved charge dynamics. Further increasing La content (Figure 6c) leads to significant lattice distortion and strong local potential fluctuations, which impair charge separation and reduce the charge carrier lifetimes. As a result, the photocatalytic activity declines sharply due to increased recombination and structural disorder.

## Conclusions

In summary, this study demonstrates that substantial enhancement in photocatalytic activity can be achieved through minimal, well-balanced codoping. We propose a trap-mediated charge transfer mechanism and highlight the complex interplay among dopant-induced electronic states, structural integrity, and charge carrier dynamics. Within a narrow compositional window, this balance is optimized to maximize performance. Beyond this range, excessive defect formation and electronic disorder dominate, ultimately suppressing the activity. These findings offer valuable insights for the rational design of codoped layered perovskite photocatalysts and deepen our understanding of dopants’ role in photocatalytic systems.

## Supplementary Material



## Notes

Synthesis and characterization methods, additional structure, electronic structure, morphology, and catalysis data and analysis (PDF)

## ∇

Hangjuan Ren - School of Chemistry, Monash University, Clayton, Victoria 3800, Australia

## ◆

S. C. E. T. deceased on May 20, 2025.
