# Supplementary material for: Synergistic Rh/La Codoping Enables Trap-Mediated Charge Separation in Layered Perovskite Photocatalysts
Source: J Am Chem Soc. 2025 Oct 13;147(42):38599–608. doi: 10.1021/jacs.5c12425 (PMC12550842; doi:10.1021/jacs.5c12425)
Supplement: Supplementary file 1 [file ja5c12425_si_001.pdf]

Supporting Information for

Synergistic Rh/La Codoping Enables Trap-Mediated Charge Separation in Layered Perovskite Photocatalysts

Mengqi Duan[1, ‡], Shuai Guo[2, ‡], Wentian Niu[1], Hangjuan Ren[1, †], Thomas Dittrich[3], Dongpei Ye[1], Lucy Saunders[4], Sarah Day[4], Veronica Celorrio[4], Diego Gianolio[4], Peixi Cong[5], Robert S. Weatherup[5], Robert Taylor[6], Songhua Cai[2,], Yiyang Li[1, *], Shik Chi Edman Tsang[1, #]*

[1] Wolfson Catalysis Centre, Department of Chemistry, University of Oxford, Oxford, OX1 3QR, U.K.

[2] Department of Applied Physics, The Hong Kong Polytechnic University, Kowloon 999077, Hong Kong SAR China

[3] Helmholtz-Zentrum Berlin für Materialien und Energie GmbH, Schwarzschildstr. 8, 12489 Berlin, Germany

[4] Diamond Light Source Ltd., Harwell Science & Innovation Campus, Didcot, OX11 0DE, U.K.

[5] Department of Materials, University of Oxford, Parks Road, Oxford, OX1 3PH, U.K.

[6] Clarendon Laboratory, Department of Physics, University of Oxford, Oxford, OX1 3PU, U.K.

This file includes:

Methods

Supporting Figures 1 – 33

Supporting Tables 1 – 13

Supporting Notes 1 – 3

Methods

Preparation of nanosheet

$KCa_{2-x}La_xNb_{3-y}Rh_yO_{1-\delta}$ (x=0, 0.04, 0.08, 0.2; y=0, 0.03, 0.15) powder was synthesized using a solid-state reaction method following a previously reported procedure.[1] The starting materials K_2SO_4 (Scientific Laboratory Supplies, 98%), $CaCO_3$ (Sigma Aldrich, 98%), Nb_2O_5(Thermo Fisher Scientific, 97%), La_2O_3 (Sigma Aldrich, 94%) and Rh_2O_3 (Alfa Aesar, 99.9%) were weighed in a mole ratio of 5: 4-2x: 3-y: x: y. The mixture was ground thoroughly in an agate mortar, transferred into an alumina crucible, then further heated to 900 ℃ at a ramp rate of 300 ℃ h^{-1}, then to 1250 ℃ at a ramp rate of 100 ℃ h^{-1} and kept at 1250 ℃ for 24 h in a muffle furnace (Carbolite Gero, RHF 16/3). The furnace was then cooled to 800 ℃ at 25 ℃ h^{-1}, followed by rapid cooling to room temperature at a rate of 300 ℃ h^{-1}. The resulting powder was washed three times to remove residual K_2SO_4 and dried in a vacuum oven at 60 ℃ overnight.

Subsequently, 2 g of the obtained powder was protonated by treating it with 350 mL of 6 M HCl solution at 60 ℃ for 18 h. After the acid exchange, the product was washed with DI water until supernatant reached a pH of 7, then dried in a vacuum desiccator at room temperature.

Exfoliation was performed by dispersing 1.5 g of the protonated product in 350 mL of tetrabutylammonium hydroxide (TBAOH, Fluorochem Ltd, 10% solution in water), where the mole ratio between the protonated product and TBAOH was 1: 1.[2] The mixture was stirred continuously for a week, with sonication for 30 minutes twice daily (CREWORKS 360w Ultrasonic Cleaner). After exfoliation, the colloidal suspension was centrifuged at 5000 rpm for 30 minutes to remove unexfoliated residues. The supernatant was further centrifuged at 14500 rpm for 1 hour to get the exfoliated nanosheet from the sediment.

Photocatalytic H_2 evolution test

Around 5 mg of the catalyst was dispersed in 10 mL of 10 vol% methanol solution and sonicated for 5 minutes in a quartz vial. The resulting suspension was transferred into a 50 mL stainless-steel autoclave with a quartz window. Then the autoclave was sealed and purged with Ar gas 15 times to remove the air inside, and then pressurized with 2 bar of Ar gas to serve as the carrier gas. The solution was continuously stirred throughout the experiment. After one hour of illumination by a Xe lamp (Sciencetech, LH-S-300X

Xenon Arc Lamp Housing), the hydrogen evolution was measured using gas chromatography (GC, Agilent technologies, 7890B GC system) equipped with a thermal conductivity detector (TCD) with N_2 as the carrier gases. A six-hour cyclic activity was monitored and the H_2 evolution amount was measured by GC every one hour. Then the reactor was purged with Ar again after each run without taking the catalysts out from the reactor.

The apparent quantum efficiency (AQE) was measured in the same reactor following the same procedure as a typical photocatalytic test. The batch reactor was this time irradiated by a 300-W Xenon lamp (Newport) equipped with band-pass filters of 300±25 nm, 330±25 nm, 365±25 nm, 405±25 nm, 437±25 nm, and 650±25 nm, respectively. Subsequently, the relevant number of incident photons was calculated from the irradiation powers at each wavelength. The AQE can be calculated by using the equation:

$$AQE(\%) = \frac{\text{Number of evolved } \mathrm{H_2} \text{ molecules} \times 2}{\text{Number of incident photons}} \times 100\% \qquad \text{Equation S1}$$

AQE measurements were repeated for at least 3 times and the average values and standard deviations were calculated.

Characterization

X-ray powder diffraction (XRD) pattern was collected using a Bruker D8 Advanced diffractometer with a Cu radiation source. No monochromator or nickel filter was used. Both the primary optics and secondary optics used 2.5° soller slits, and a knife antiscatter was positioned 2mm above the sample. The instrument was operated in step mode with a step size of 0.01 ° and 0.25 s per step at 40 kV and 25 mA.

Synchrotron XRD (SXRD) patterns were collected at Beamline I11 of the Diamond Light Source Ltd, UK (DLS). The incident energy was specified as 15 keV with a wavelength of 0.823809 Å and an angular zero-error of 0.00458410 ° in 2θ, calibrated using a NIST silicon standard. Samples were loaded into borosilicate capillaries with a 0.5 mm diameter and analyzed by Debye-Sherrer geometry using a multi-analyzer crystal (MAC) diffractometer or position sensitive detector (PSD) at room temperature.

X-ray fluorescence (XRF) measurement was collected using a Rigaku ZSX Primus IV. A sensitivity library derived from standards run in the factory was used for semi-quantitative elemental analysis.

X-ray photoelectron spectroscopy (XPS) measurement was performed using a Thermo NEXSA XPS fitted with a monochromatic Al kα X-ray source (1486.7 eV), a spherical sector analyzer, and 3 multichannel resistive plate 128-channel delay line detectors. All data was recorded at 72W with an X-ray beam size of 400 x 200 µm. Survey scans were recorded at a pass energy of 160 eV, and higher resolution narrow scans were recorded at a pass energy of 20 eV. Electronic charge neutralization was achieved using a Dual-beam low-energy electron/ion source (Thermo Scientific FG-03). Ion gun current = 150 µA. Ion gun voltage = 45 V. All sample data was recorded at a pressure below 10^{-8} Torr and a room temperature of 294 K. The charge correction was done using C-C peak of adventitious carbon at 284.8 eV in C 1s spectra. XPS data was analyzed using CasaXPS v2.3.18PR1.0. Peaks were fitted with a Shirley background prior to component analysis. Line shapes of LA (1.2,3,2) were used to fit Rh^0 components and line shapes of GL (30) were used to fit other components. The XPS fitting was performed with reference to data from several databases, including NIST, ThermoFisher, and XPSfitting. Ultraviolet photoelectron spectroscopy (UPS) analysis was performed on the same instrument with a He Ⅰ source (21.22 eV).

X-ray absorbance spectra (XAS) at the Rh *K*-edge were collected in fluorescence mode with a Si (311) monochromator at beamline B18 at Diamond Light Source, UK. Transmission La L_3-edge XAFS spectra were collected with a laboratory-based easyXAFS300+ spectrometer (easyXAFS, WA, US). X-rays are generated with a liquid-cooled Mo anode X-ray tube, before monochromation by a Si (331) spherically bent crystal analyzer. A helium-filled box with polyimide windows is placed in the beam path for better X-ray transparency, while a steel plate with a 9 × 3 mm slot is placed after each sample. The transmitted intensity is measured with a silicon drift detector (KETEK, Munich, Germany) placed behind the sample. Each acquisition was performed over 40 min, and 110 scans were collected for each sample. La_2O_3 reference spectra were collected for energy calibration. Data pre-processing was performed with the EasyXANES package to convert the measured intensity into linear attenuation coefficient, µ.

UV-vis diffuse reflectance spectroscopy (UV-vis DRS) was performed using a Shimadzu UV-2600 spectrophotometer equipped with an integrating sphere in single-scan mode at medium speed. A disk of pressed $BaSO_4$ powder was used as the 100% reflecting reference.

Scanning electron microscopy (SEM) images were taken on a JEOL JSM-6610LV microscope operated at 20 kV with a working distance of 11 mm. Samples were sprinkled on carbon tapes attached to stainless steel stubs.

High-angle annular dark-field scanning transmission electron microscope (HAADF-STEM) images were taken on a double spherical aberration-corrected (Spectra 300, Thermofisher, USA; equipped with a field emission gun) S/TEM system at 300 kV electron beam accelerating voltage. The electron probe convergence angle was 24.5 mrad, and the angular range of the HAADF detector was from 79.5 mrad to 200 mrad. The dwell time of each pixel for HAADF image acquisition is 6 µs. The beam current was reduced to below 2 pA, thus reducing the electron dose below the threshold values for radiation damage. STEM samples were prepared by dispersing 1 mg of powder dispersed in 5 ml of deionized water and sonicated for 30 minutes.

Atomic force microscopy (AFM) was conducted on a Dimension 5000 microscope. Samples were prepared by dispersing 1 mg sample in 2 ml ethanol, sonicated for 30 minutes, and then spin-coated onto a 1 cm x 1 cm silicon wafer (Sigma Aldrich, <100>, P-type) using a spin coater (Laurell Technologies WS-650MZ-23NPP/LITE).

Continuous wave EPR (CW-EPR) data were collected on a Bruker EMXmicro X band spectrometer with a Premium bridge, utilizing a Bruker ER4122SHQE-W1 resonator, at the Centre for Advanced ESR (CAESR) of the Department of Chemistry, University of Oxford.

Time resolved photoluminescence (TRPL) spectra were collected from a bespoke micro-photoluminescence setup in which a Ti-sapphire laser (λ = 266 nm, pulse duration = 150 fs, repetition rate = 76 MHz) was directed onto the sample. The TRPL spectra were fitted with a bi-exponential function: $y = A_1 \cdot \exp(-x/\tau_1) + A_2 \cdot \exp(-x/\tau_2) + y_0$.

Surface photovoltage spectroscopy (SPV) was measured on a home-made setup with a lock-in amplifier (EG&G5210), high-impedance buffer, quartz-prism monochromator (SPM2), a Xe-lamp, and an optical copper. 12 mg of exfoliated sample was dispersed in 2.5 mL ethanol and sonicated for 15 minutes following

a cycle of 3 seconds on and 3 seconds off, with an amplitude of 30%. The catalyst ink was then drop casted on ITO glass.

The Brunauer-Emmett-Teller (BET) surface area was obtained from N_2 adsorption/desorption isotherms at −196°C and analyzed by a Micromeritics TriStar II Plus instrument. All sorption isotherms were obtained using ultrahigh purity nitrogen gas (99.999%) to calculate the surface area of the desired sample. The tested sample (approximately 100 mg) was loaded into a sample cell and pre-treated under vacuum of 10^{-5} Torr at 110°C for 24 h.

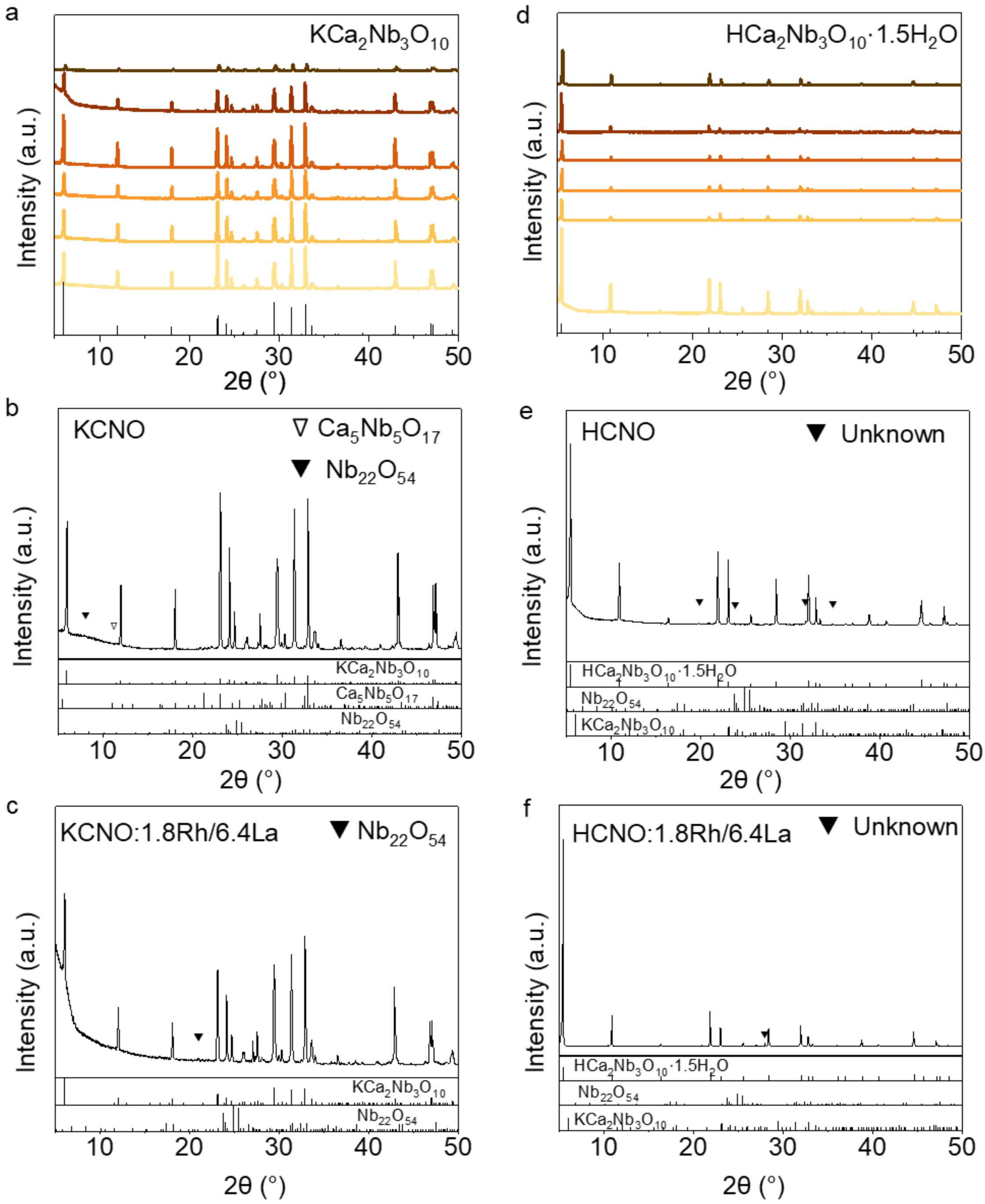

Supporting Figure 1. XRD patterns of precursors. (a) XRD patterns of undoped and doped KCNO. From bottom to up, KCNO, KCNO:0.2Rh, KCNO:0.2Rh/1.3La, KCNO:0.6Rh/2.2La, KCNO:1.8Rh/6.4La, and KCNO:1.3La. (b) and (c) The detailed XRD patterns of undoped KCNO and KCNO:1.8Rh/6.4La. (d) XRD patterns of undoped and doped HCNO. From bottom to up, HCNO, HCNO:0.2Rh, HCNO:0.2Rh/1.3La, HCNO:0.6Rh/2.2La, HCNO:1.8Rh/6.4La, and HCNO:1.3La. (e) and (f) XRD patterns of undoped HCNO and HCNO:1.8Rh/6.4La.

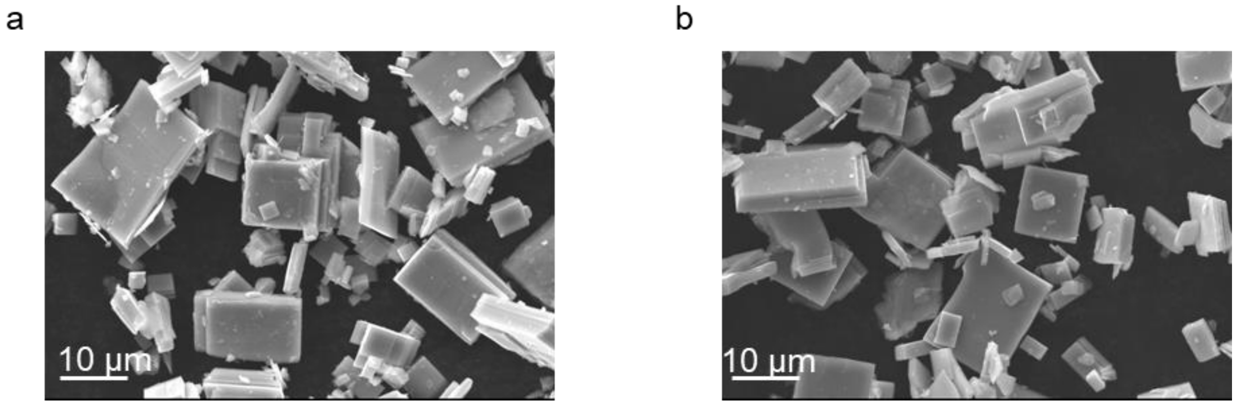

Supporting Figure 2. SEM images of (a) HCNO:0.2Rh and (b) HCNO:0.2Rh/1.3La.

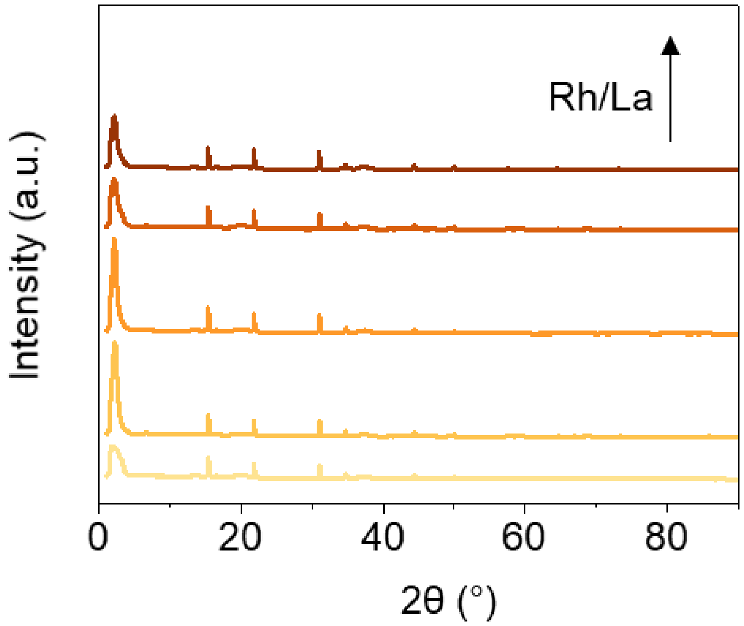

Supporting Figure 3. SXRD pattern of undoped and doped TCNO collected using PSD. From Bottom to top: TCNO, TCNO:0.2Rh, TCNO:0.2Rh/1.3La, TCNO:0.6Rh/2.2La, TCNO:1.8Rh/6.4La.

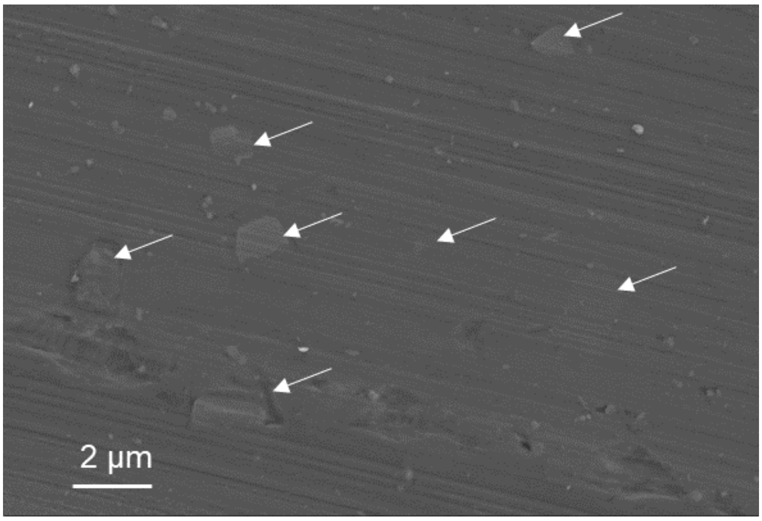

Supporting Figure 4. SEM image of TCNO nanosheets.

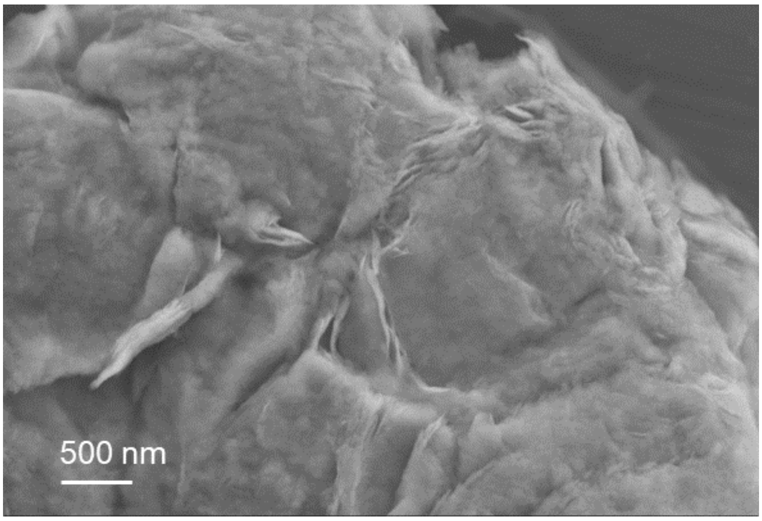

Supporting Figure 5. SEM image showing aggregation of dried TCNO nanosheets

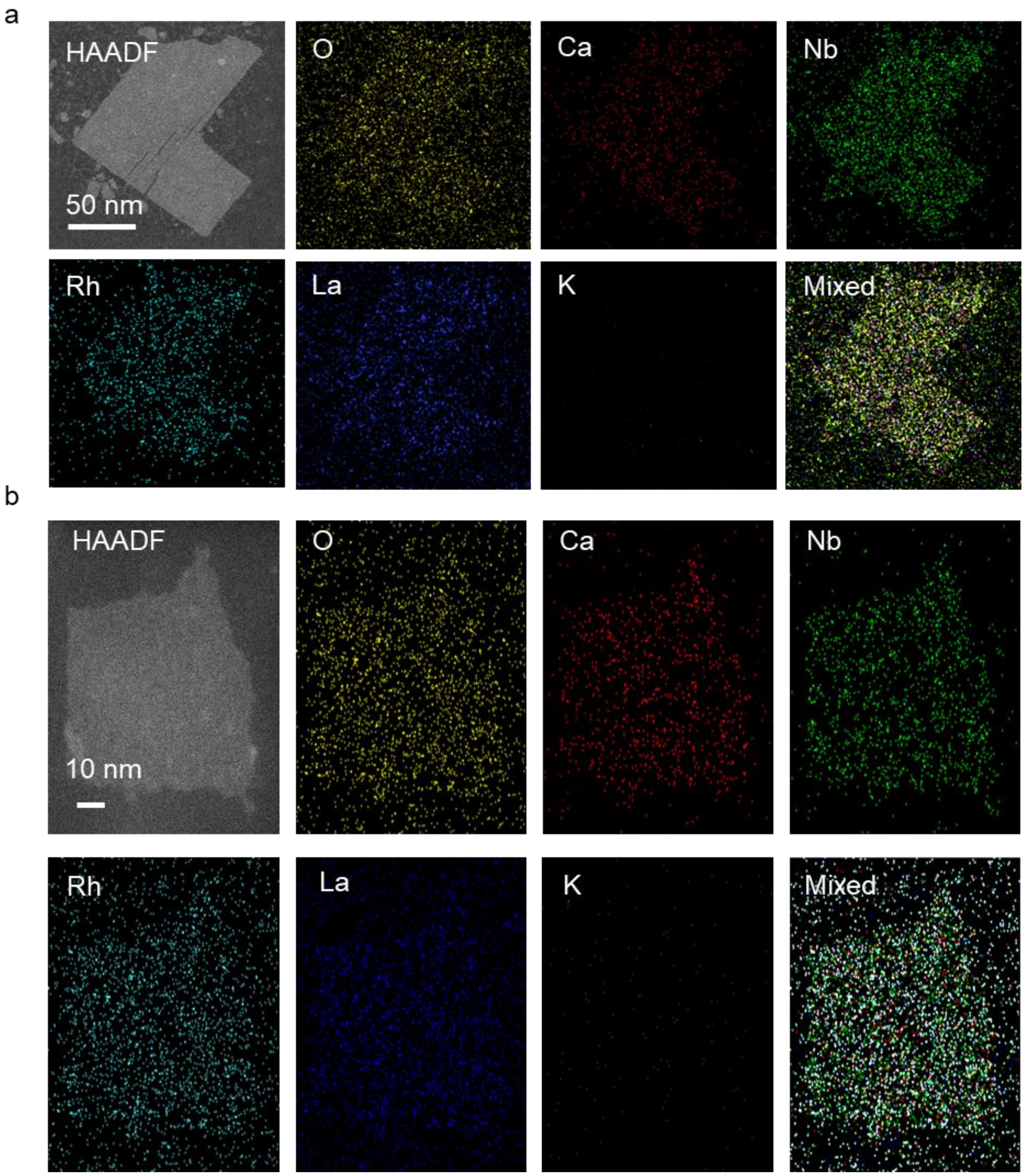

Supporting Figure 6. STEM-EDX mapping of TCNO:0.2Rh/1.3La in (a) Region 1 and (b) Region 2.

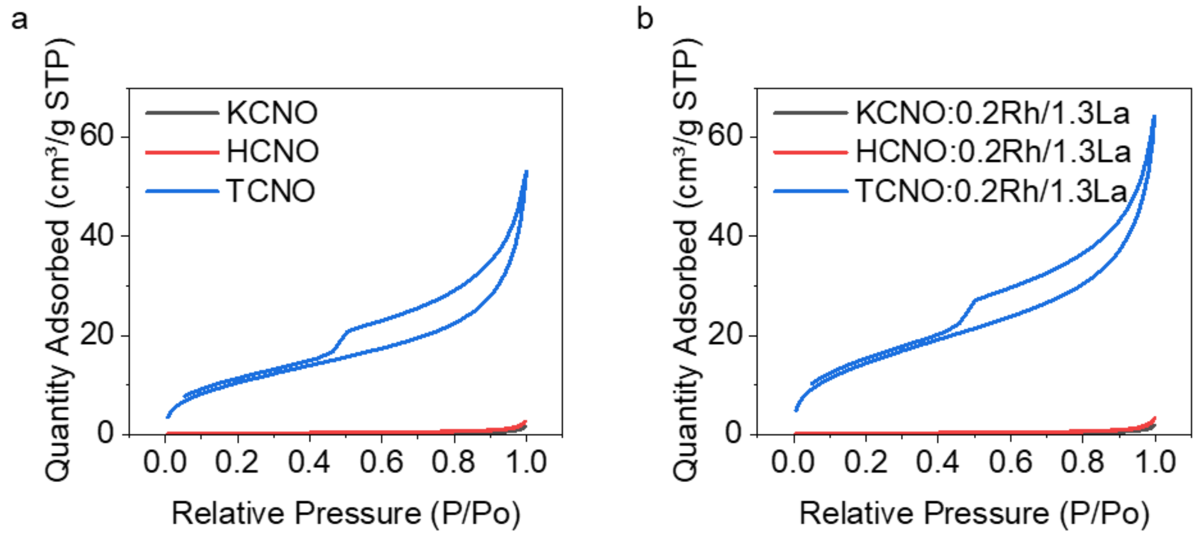

Supporting Figure 7. N_2 adsorption/desorption isotherm comparison between bulk layered perovskite and exfoliated layered perovskite nanosheets. (a) undoped layered perovskite. (b) 0.2Rh/1.3La codoped layered perovskite.

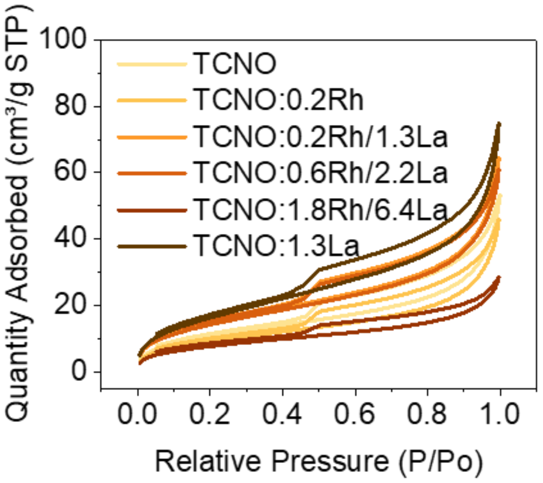

Supporting Figure 8. N_2 adsorption/desorption isotherms of doped and undoped exfoliated layered perovskite nanosheets.

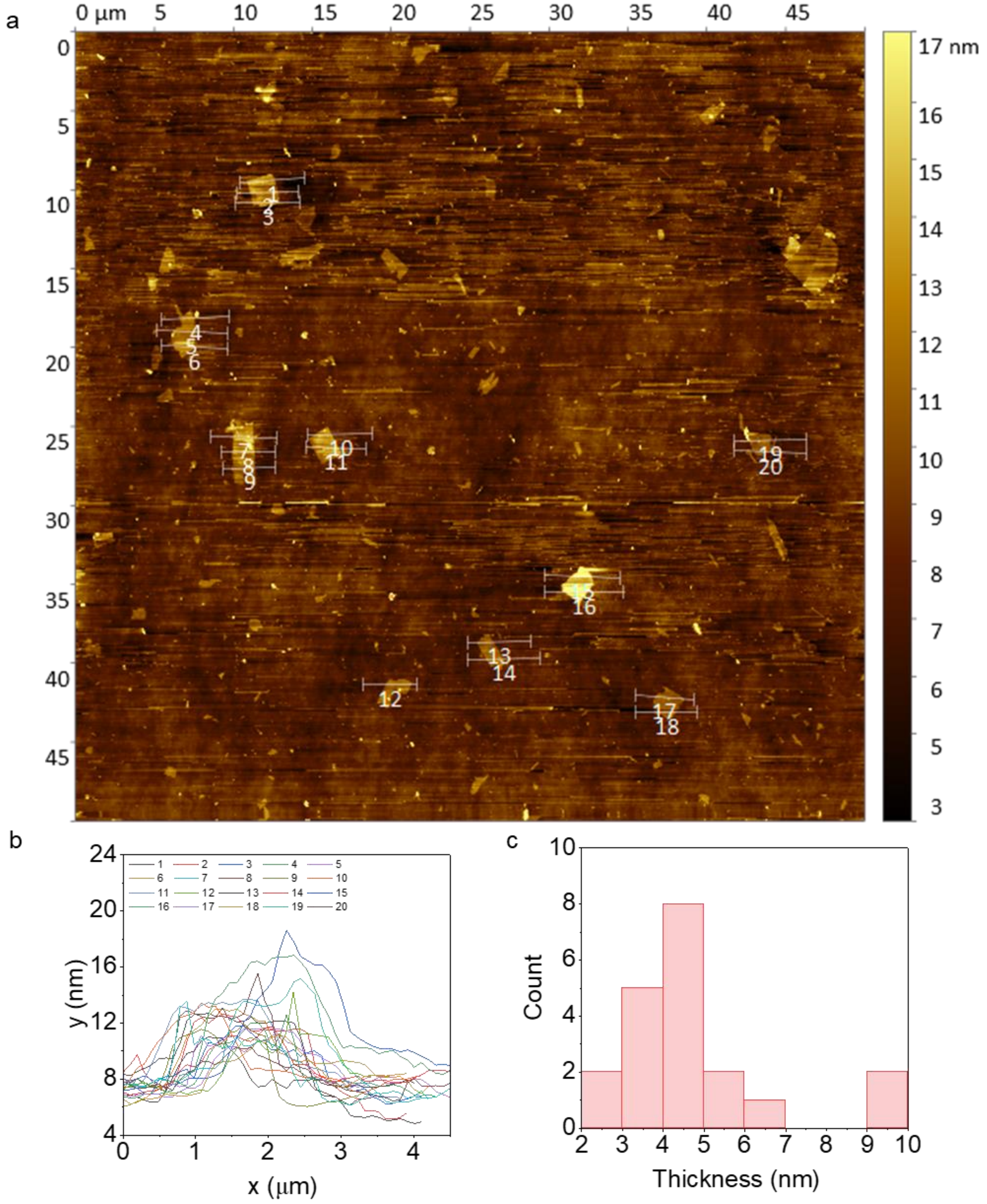

Supporting Figure 9. Thickness of TCNO:0.2Rh/1.3La. (a) AFM image of TCNO:0.2Rh/1.3La. (b) The height profile of nanosheets. (c) The thickness distribution of nanosheets in (a). The single perovskite slab was around 3 nm in literature, presumably due to the adsorption of water molecules and ammonium ions as well as other systematic factors in the AFM experiments.[3]

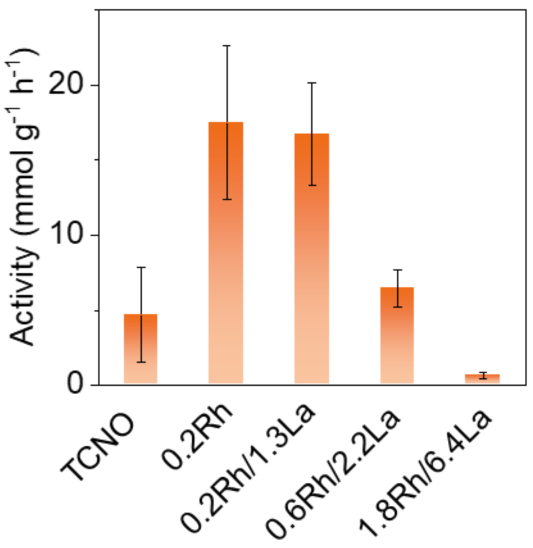

Supporting Figure 10. Photocatalytic HER activity of undoped and doped TCNO synthesized and tested from different batches.

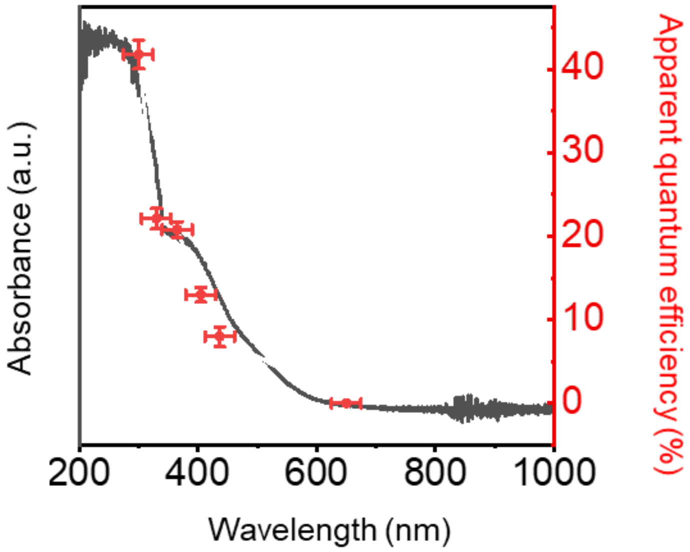

Supporting Figure 11. Wavelength-dependent apparent quantum efficiency (AQE) measurements on TCNO:0.2Rh/1.3La.

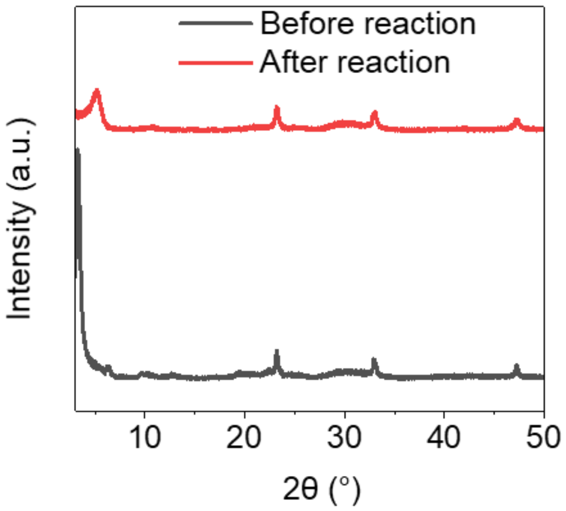

Supporting Figure 12. XRD of TCNO:0.2Rh/1.3La before and after six-hour reaction. The in-plane diffraction peaks remained identical, while (001) peak shifted toward higher angle, indicating that TBA^+ ions were degraded and replaced by protons, leading to a reduced interlayer spacing.[4]

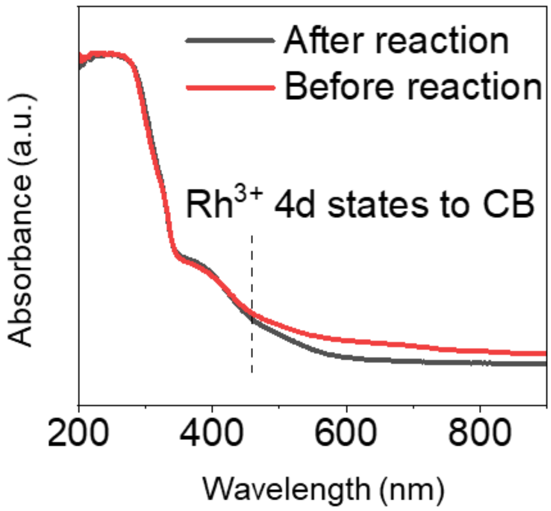

Supporting Figure 13. UV-vis DRS of TCNO:0.2Rh/1.3La before and after six-hour reaction. The dashed line indicates the absorption edge due to electronic transitions from Rh^{3+} 4d states to conduction band (CB).

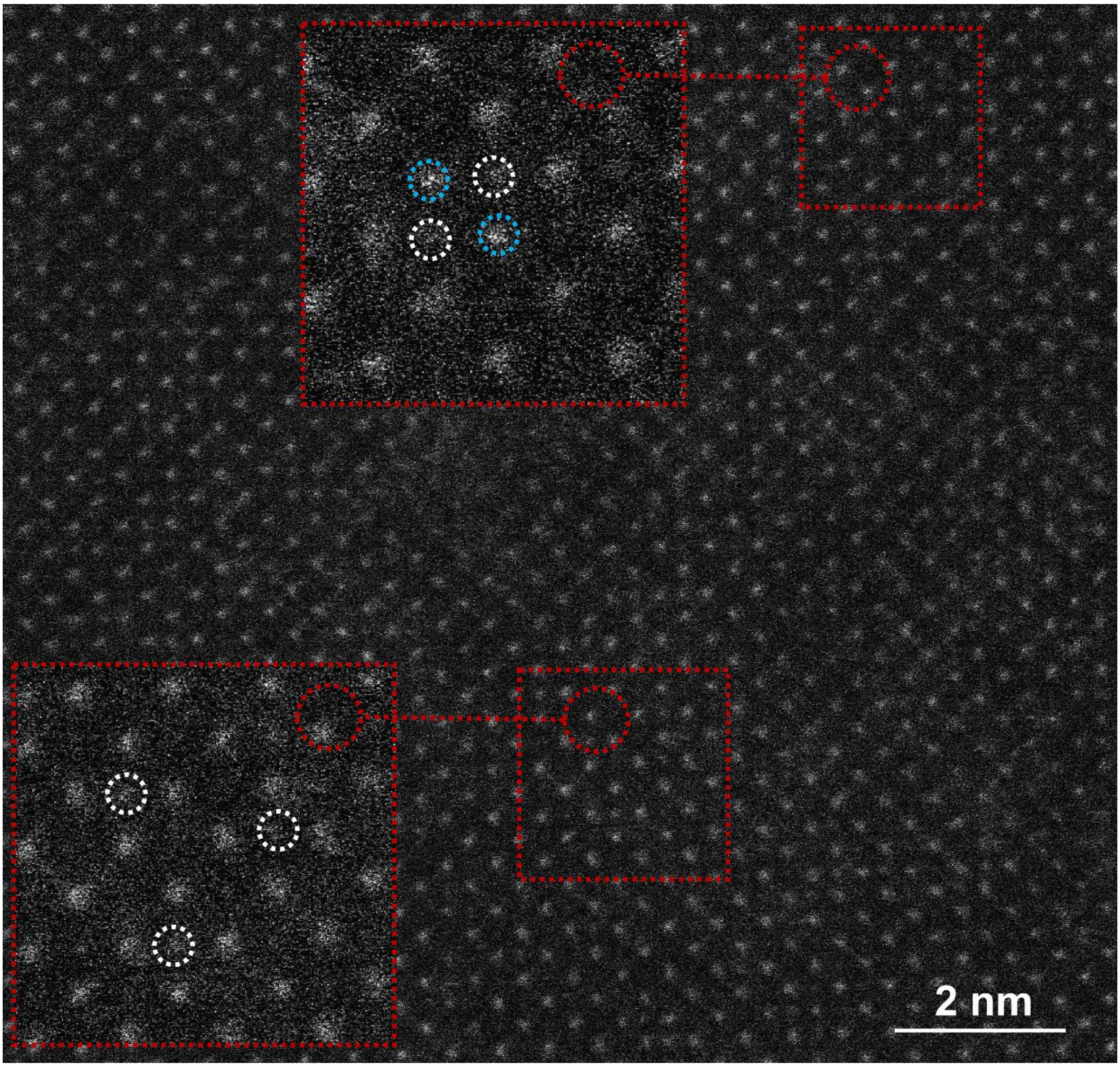

Supporting Figure 14. High resolution HAADF-STEM of TCNO:0.2Rh/1.3La after six-hour cyclic reaction. Rh substituted at B site (white circles) and La substituted at A site (blue circles).

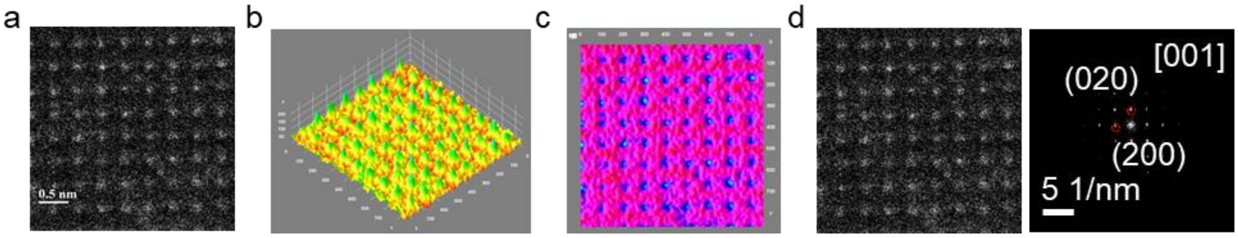

Supporting Figure 15. High resolution HAADF-STEM study of TCNO:0.2Rh/1.3La after six-hour cyclic reaction. (a) Atomic-resolution HAADF-STEM image of TCNO:0.2Rh/1.3La and corresponding ImageJ-processed representations: (b) a 3D atomic model for height determination and (c) an atomic contrast color-coded top view for atomic position mapping. Rh substituted at B site (white circles and arrows) and La substituted at A site (blue circles and arrows). (d) FFT pattern through [001] zone axis.

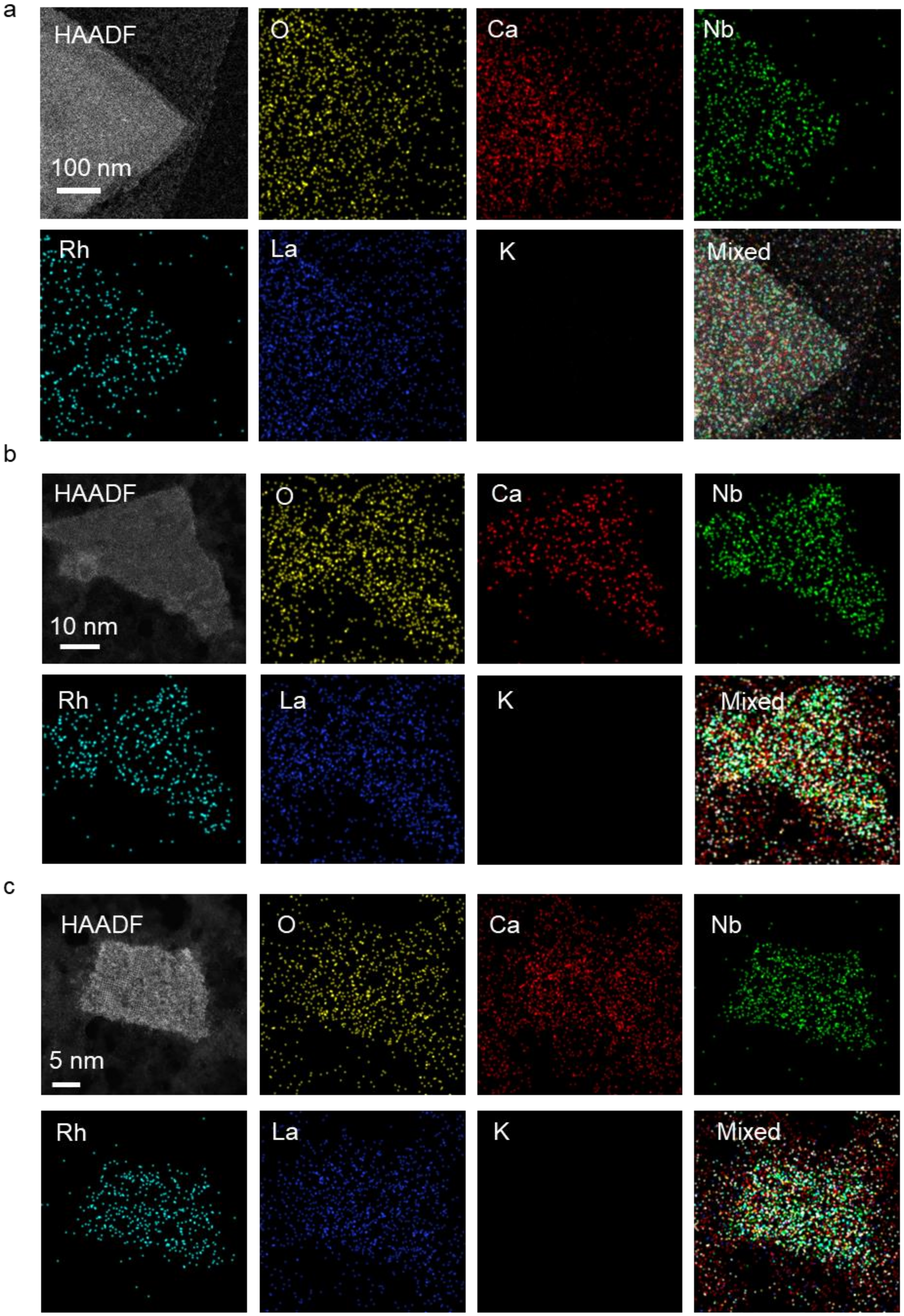

Supporting Figure 16. STEM-EDX mapping of TCNO:0.2Rh/1.3La after reaction in (a) Region 1, (b) Region 2, and (c) Region 3.

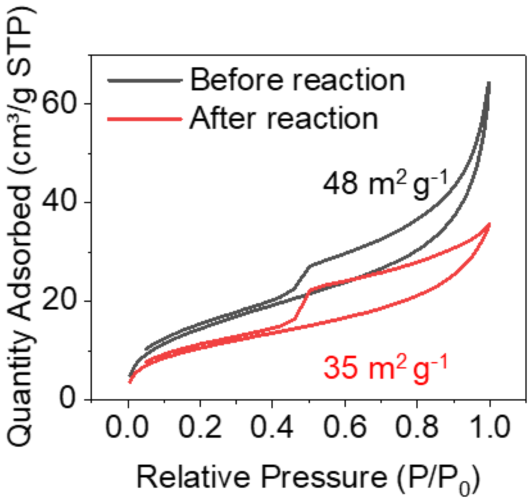

Supporting Figure 17. BET of TCNO:0.2Rh/1.3La before and after 6-hour reaction. The BET surface area decreased from 48 m^2 g^{-1} to 35 m^2 g^{-1} after reaction.

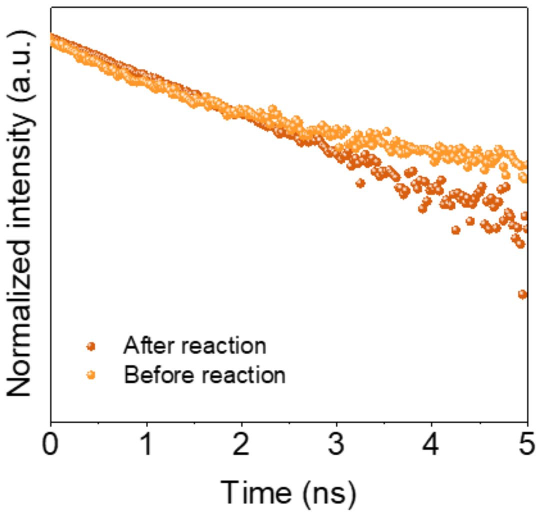

Supporting Figure 18. Normalized TRPL spectra of TCNO:0.2Rh/1.3La before and after reaction.

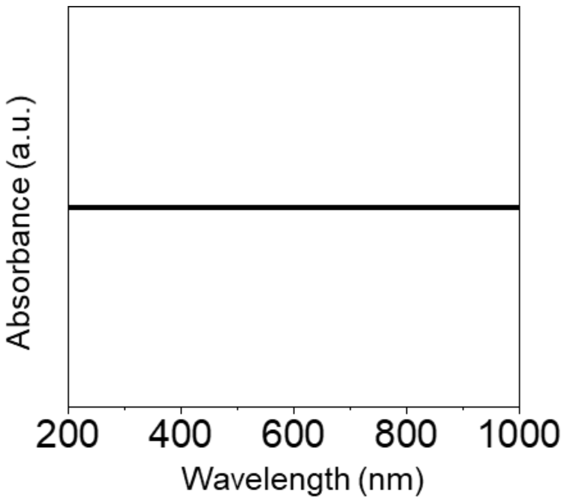

Supporting Figure 19. UV-vis absorbance spectrum of the reaction solution. After photocatalyst removal by centrifugation at 14,500 rpm for 2 h, the solution was concentrated by heating at 100 °C and then treated with 1 M HNO_3. If present, Rh species would be oxidized to Rh(III) and form a $Rh(NO_3)_3$ complex, which exhibits a characteristic absorption peak at 200 – 210 nm.

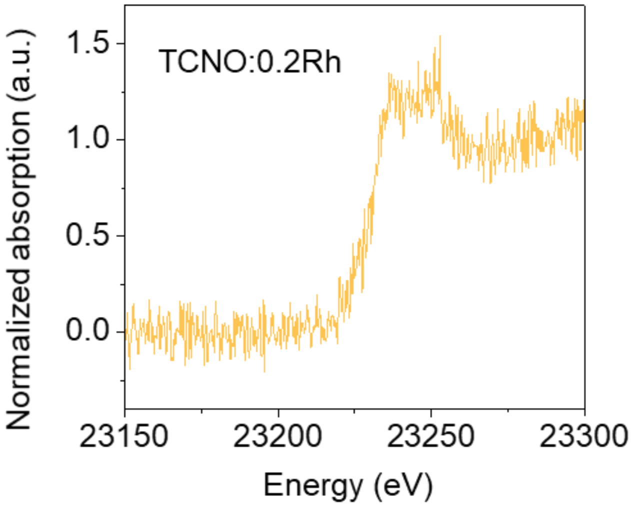

Supporting Figure 20. Rh *K*-edge XANES spectrum of TCNO:0.2Rh.

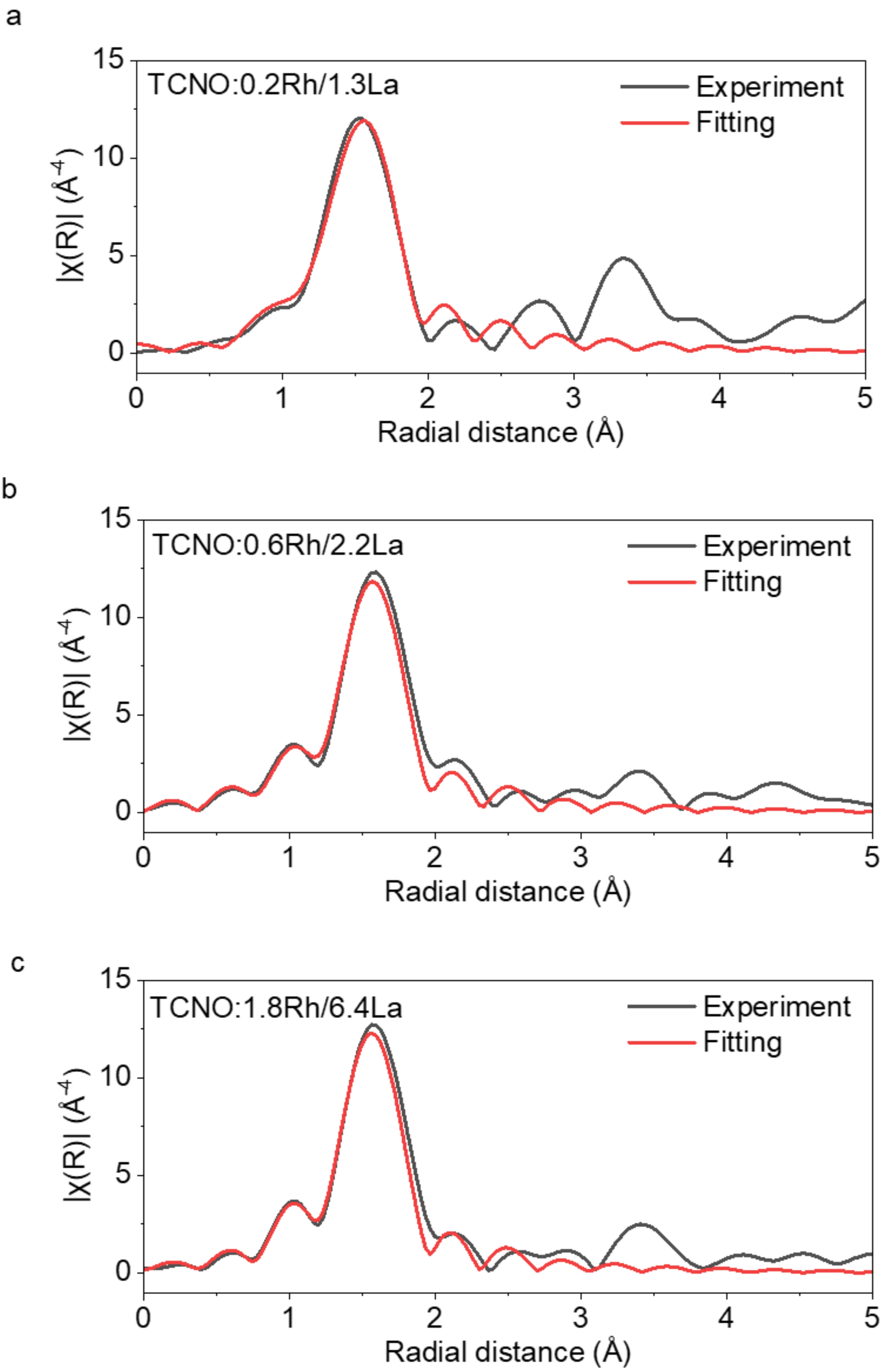

Supporting Figure 21. Fourier transformed EXAFS spectra for Rh *K*-edge. (a) TCNO:0.2Rh/1.3La. (b) TCNO:0.6Rh/2.2La. (c) TCNO:1.8Rh/6.4La. The fitting window was between 1.0 and 2.2 Å.

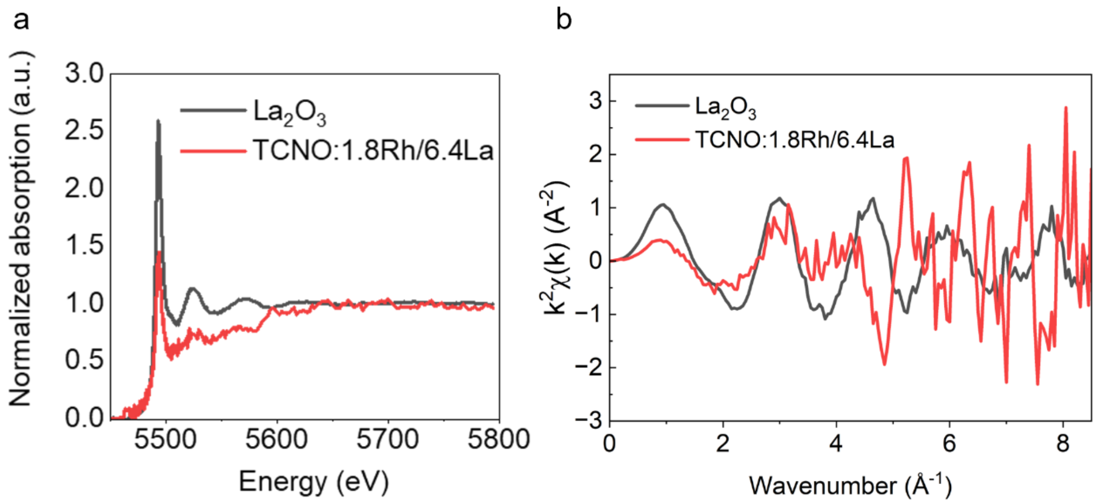

Supporting Figure 22. (a) La L_3-edge XANES spectra of TCNO:1.8Rh/6.4La compared with La_2O_3. (b) k^2-weighted La L_3-edge EXAFS spectra of TCNO:1.8Rh/6.4La compared with La_2O_3.

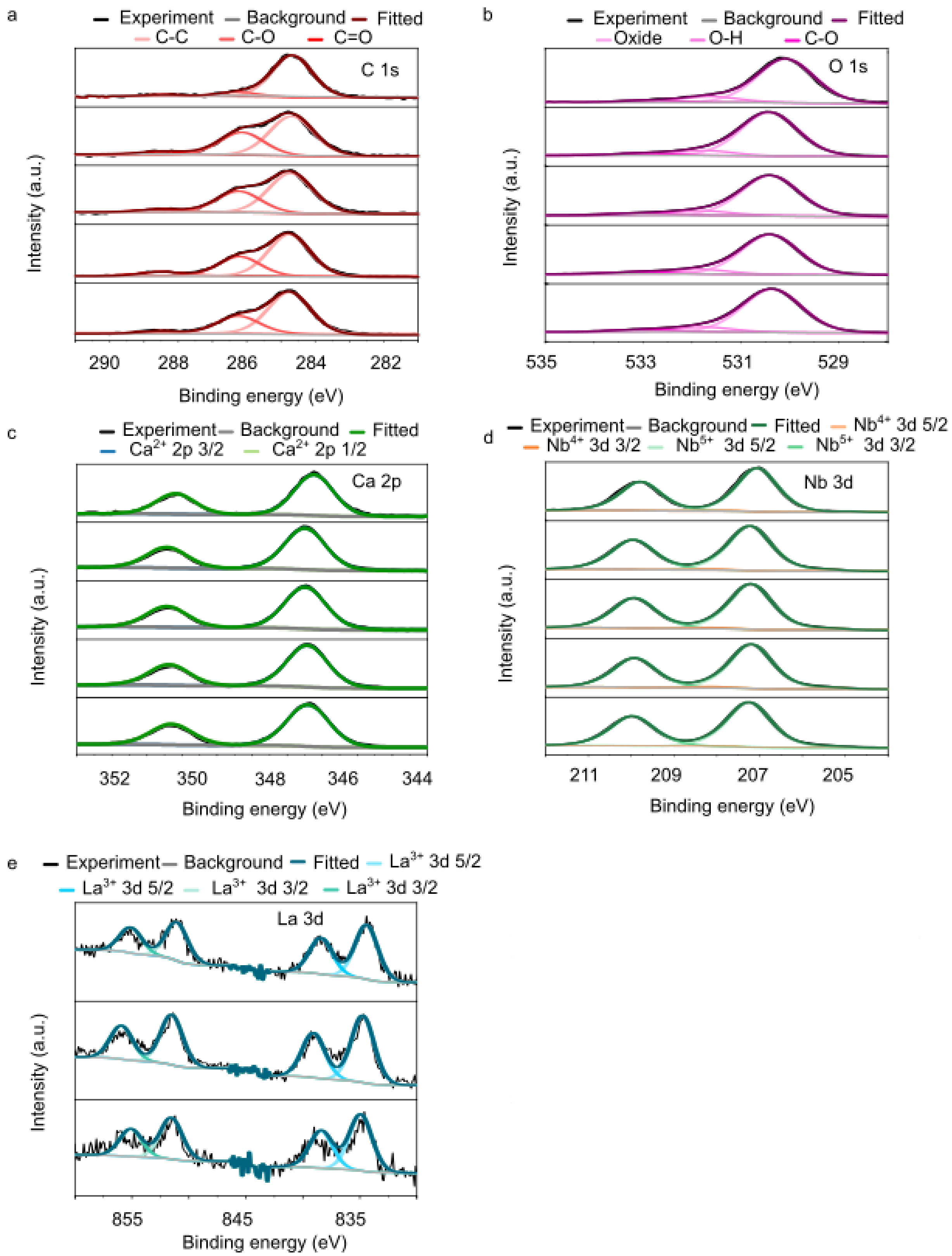

Supporting Figure 23. Narrow scan XPS spectra of doped and undoped HCNO. (a) C 1s. (b) O 1s. (c) Ca 2p. (d) Nb 3d. (e) La 3d. The samples from bottom to top in a-d are TCNO, TCNO:0.2Rh, TCNO:0.2Rh/1.3La, TCNO:0.6Rh/2.2La, and TCNO:1.8Rh/6.4La. The samples in e from bottom to top are TCNO:0.2Rh/1.3La, TCNO:0.6Rh/2.2La, and TCNO:1.8Rh/6.4La. Analysis and discussion were given in Supporting Note 1.

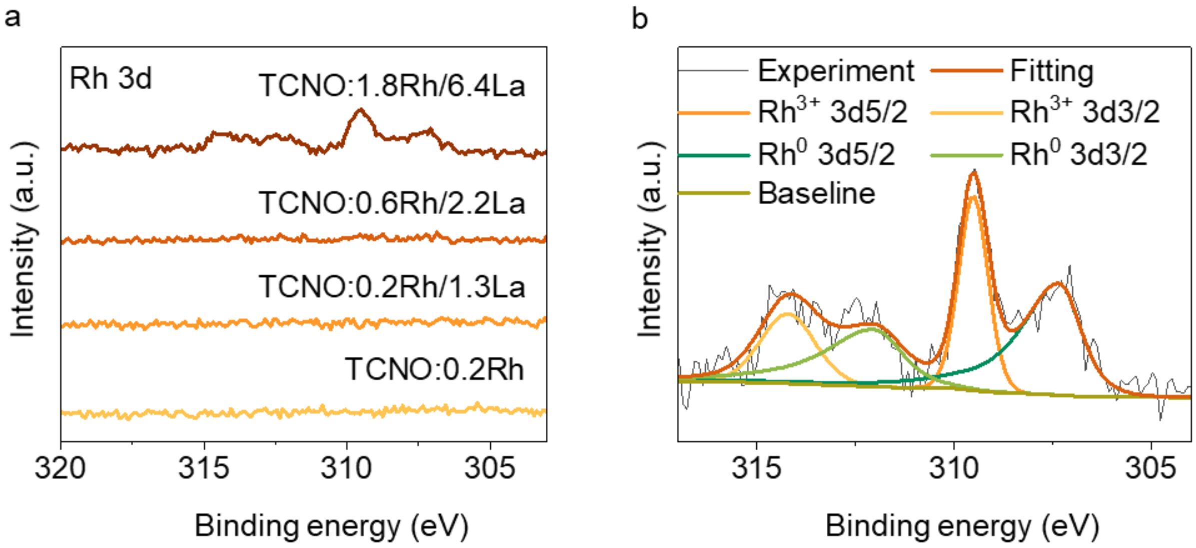

Supporting Figure 24. XPS analysis of doped TCNO nanosheets. (a) Narrow-scan Rh 3d spectra of undoped and doped TCNO. (b) Fitted XPS Rh 3d spectrum of TCNO:1.8Rh/6.4La for identification of Rh species.

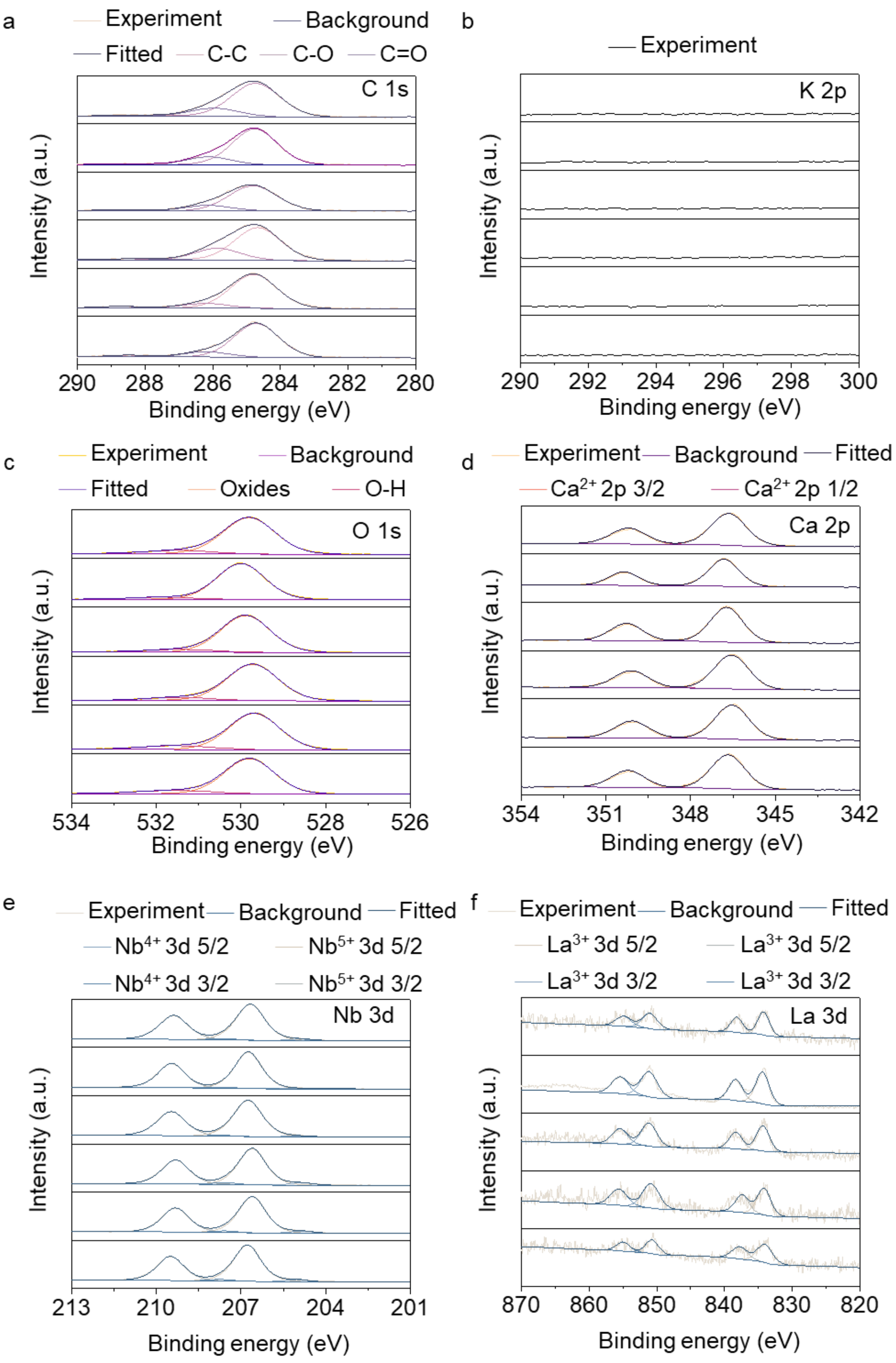

Supporting Figure 25. Narrow-scan XPS spectra of doped and undoped TCNO. (a) C 1s. (b) K 2p. (c) O 1s. (d) Ca 2p. (e) Nb 3d. (f) La 3d. The samples from bottom to top in (a) - (e) are TCNO, TCNO:0.2Rh, TCNO:0.2Rh/1.3La, TCNO:0.6Rh/2.2La, TCNO:1.8Rh/6.4La, and TCNO:1.3La. The samples in f from bottom to top are TCNO:0.2Rh/1.3La, TCNO:0.6Rh/2.2La, TCNO:1.8Rh/6.4La, and TCNO:1.3La.

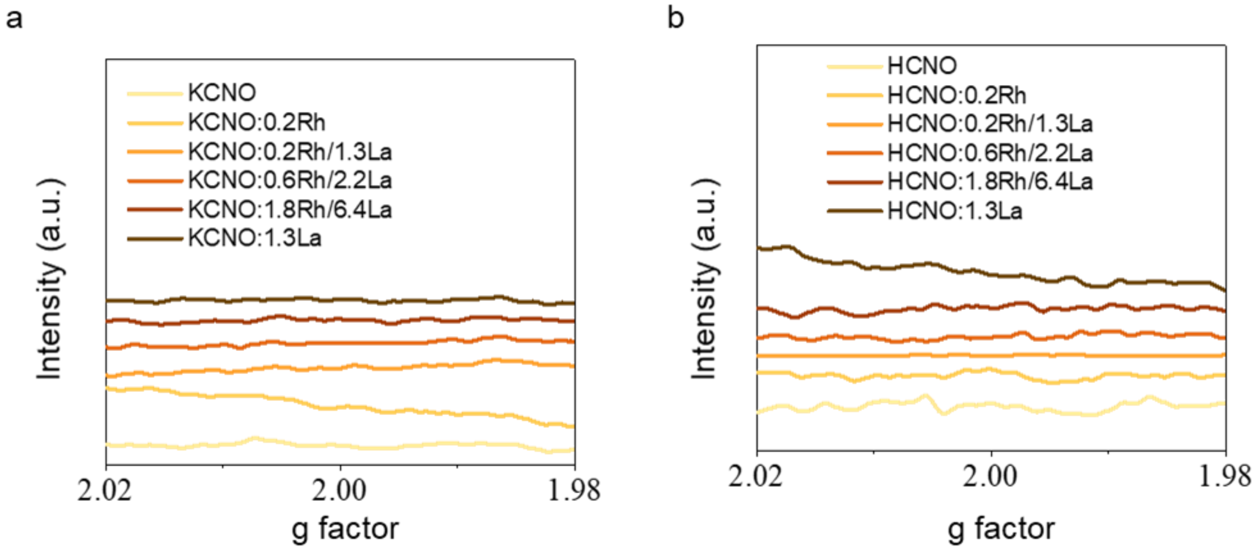

Supporting Figure 26. EPR spectra of (a) undoped and doped KCNO, and (b) undoped and doped HCNO.

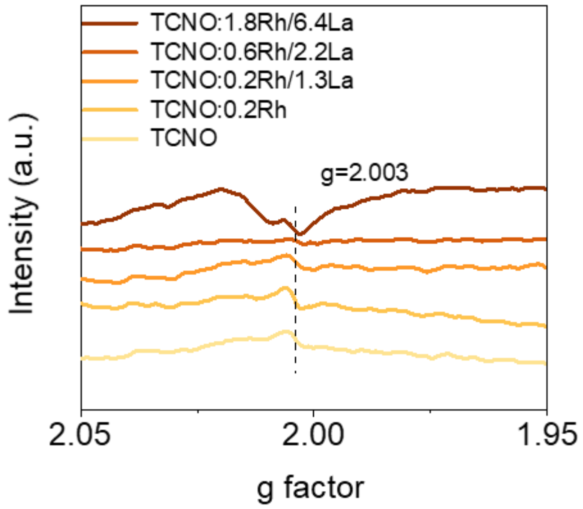

Supporting Figure 27. EPR spectra of undoped and doped TCNO.

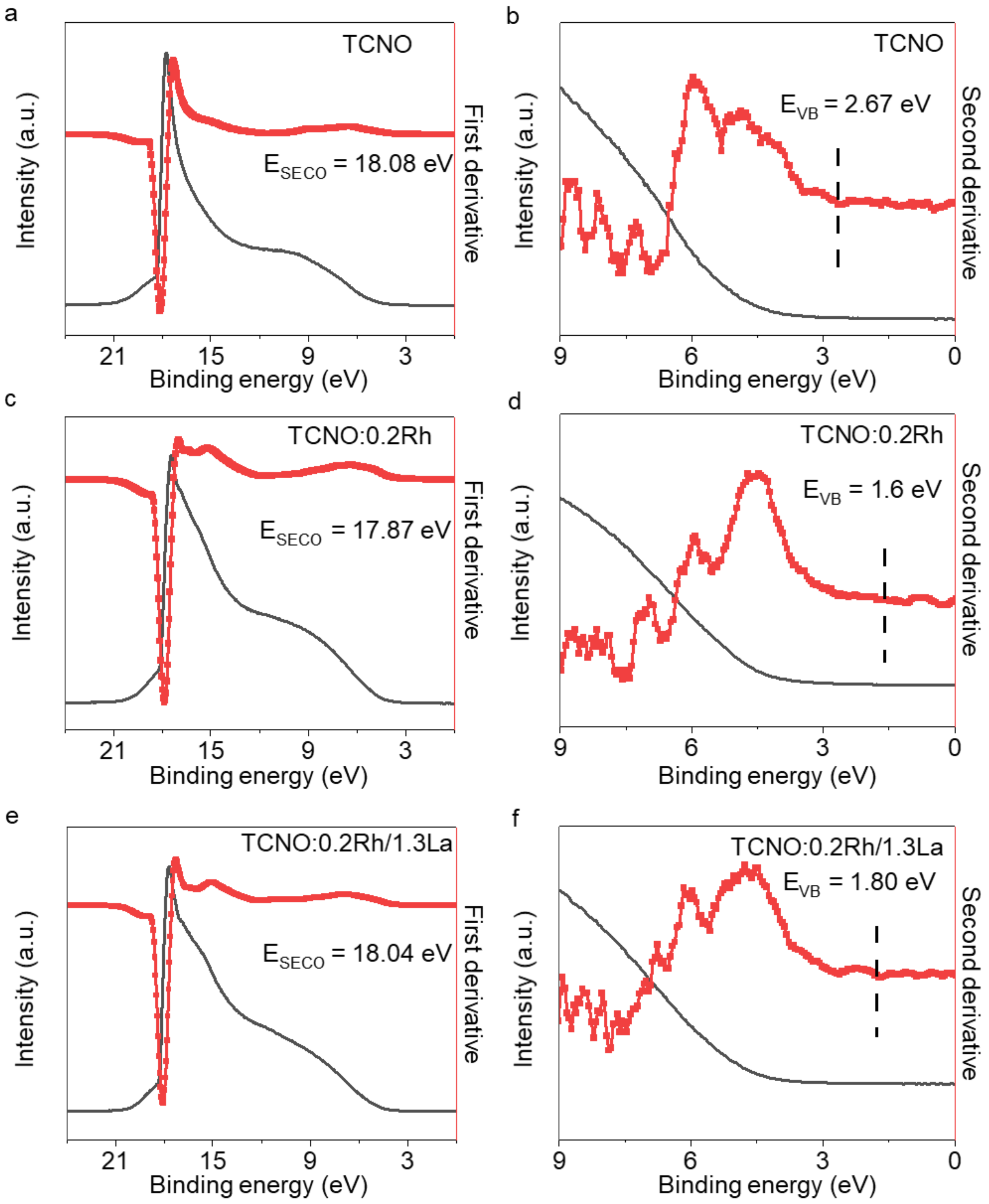

a
TCNO
E_{SECO} = 18.08 eV
Intensity (a.u.)
First derivative
Binding energy (eV)
21
15
9
3
b
TCNO
E_{VB} = 2.67 eV
Intensity (a.u.)
Second derivative
Binding energy (eV)
9
6
3
0
c
TCNO:0.2Rh
E_{SECO} = 17.87 eV
Intensity (a.u.)
First derivative
Binding energy (eV)
d
TCNO:0.2Rh
E_{VB} = 1.6 eV
Intensity (a.u.)
Second derivative
Binding energy (eV)
e
TCNO:0.2Rh/1.3La
E_{SECO} = 18.04 eV
Intensity (a.u.)
First derivative
Binding energy (eV)
f
TCNO:0.2Rh/1.3La
E_{VB} = 1.80 eV
Intensity (a.u.)
Second derivative
Binding energy (eV)

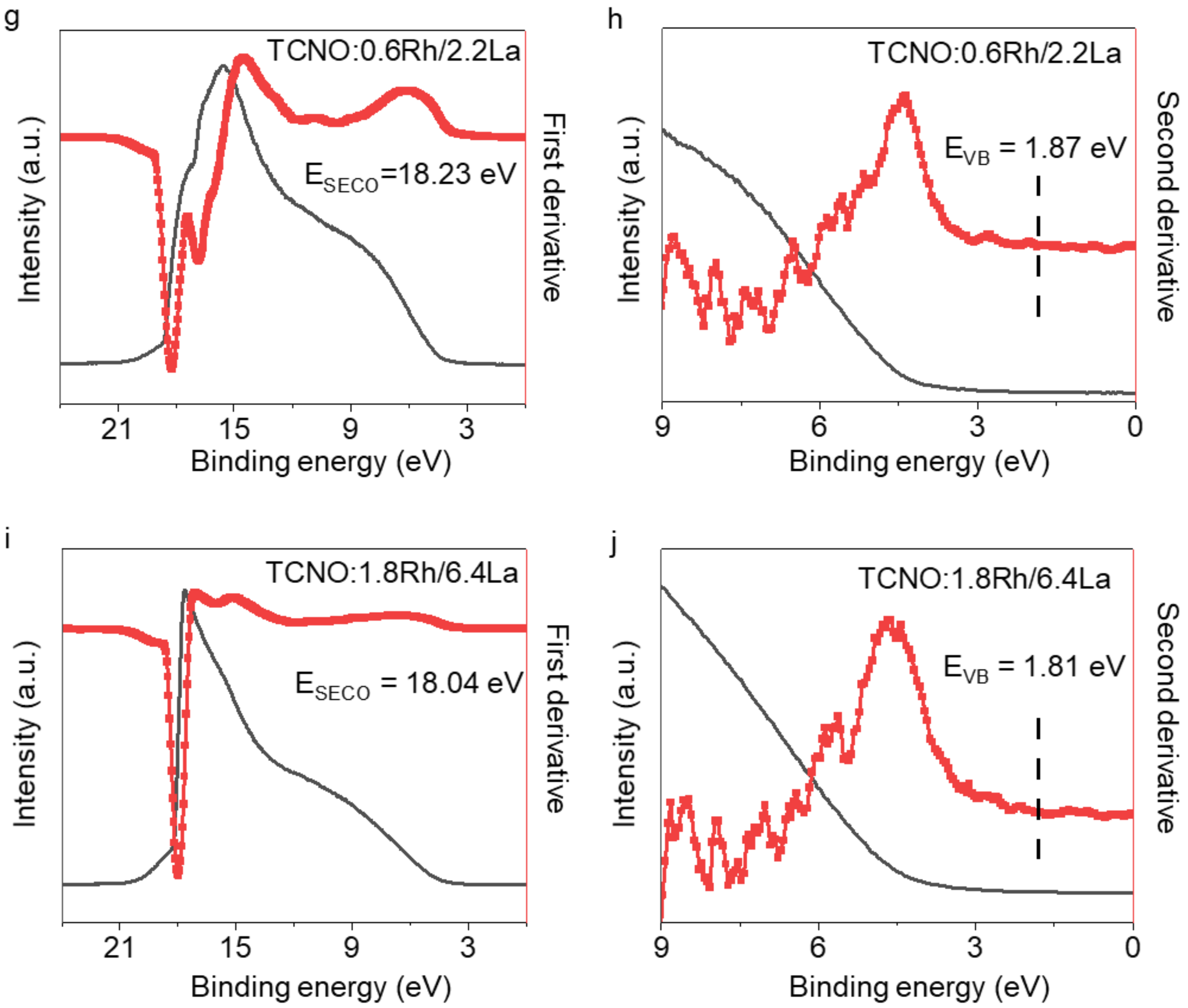

Supporting Figure 28. UPS analysis of undoped and doped TCNO. (a) and (b) TCNO. (c) and (d) TCNO:0.2Rh. (e) and (f) TCNO:0.2Rh/1.3La. g and h, TCNO:0.6Rh/2.2La. (i) and (j) TCNO:1.8Rh/6.4La. The secondary electron cut off is determined using first derivative method. The valence band maximum is determined using secondary derivative method.[5]

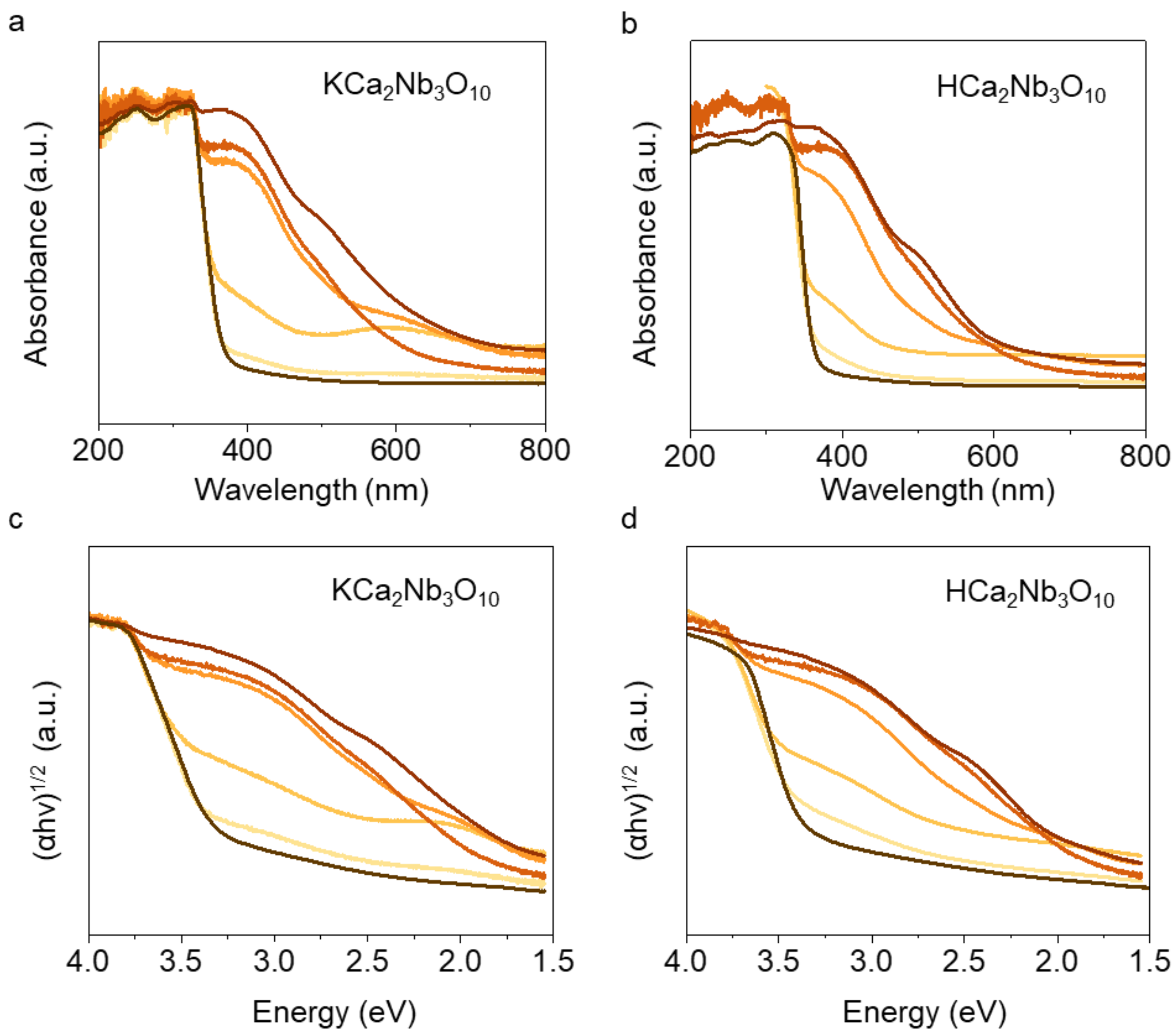

Supporting Figure 29. UV-vis DRS of bulk layered perovskites. (a) UV-vis DRS spectra and (c) Tauc plot of undoped and doped KCNO. (Golden yellow curve: TCNO; Orange curve: TCNO:0.2Rh; Burnt-Orange curve: TCNO:0.2Rh/1.3La; Orange-Brown curve: TCNO:0.6Rh/2.2La; Medium-Brown curve: TCNO:1.8Rh/6.4La; Dark-Brown curve: TCNO:1.3La). (b) UV-vis DRS spectra and (d) Tauc plot of undoped and doped HCNO. (Golden yellow curve: HCNO; Orange curve: HCNO:0.2Rh; Burnt-Orange curve: HCNO:0.2Rh/1.3La; Orange-Brown curve: HCNO:0.6Rh/2.2La; Medium-Brown curve: HCNO:1.8Rh/6.4La; Dark-Brown curve: HCNO:1.3La)

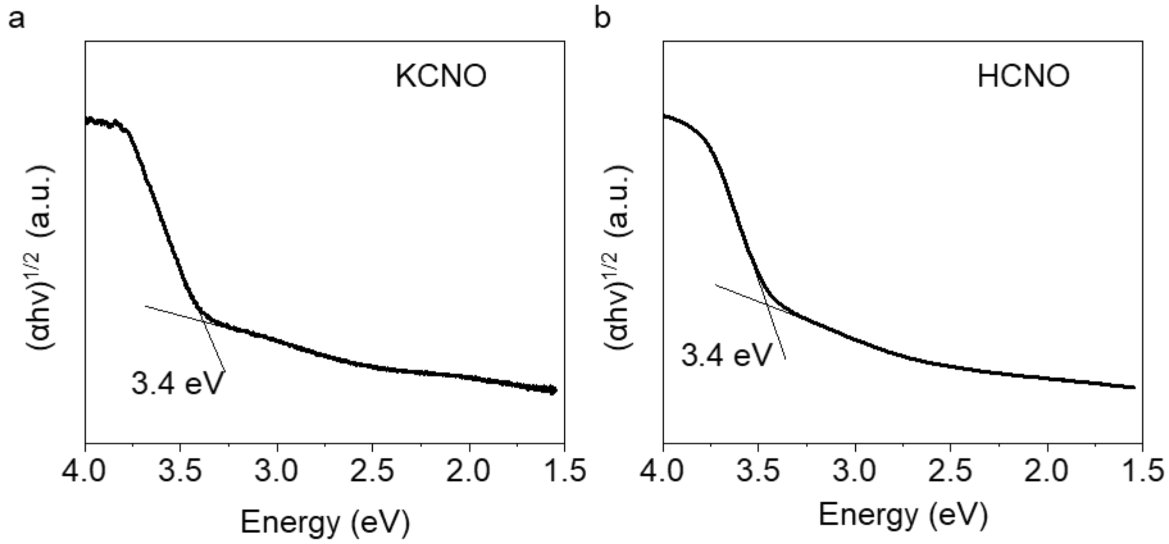

Supporting Figure 30. The absorbance edge determined in the Tauc plot converted from UV-vis DRS spectra of (a) KCNO, and (b) HCNO.

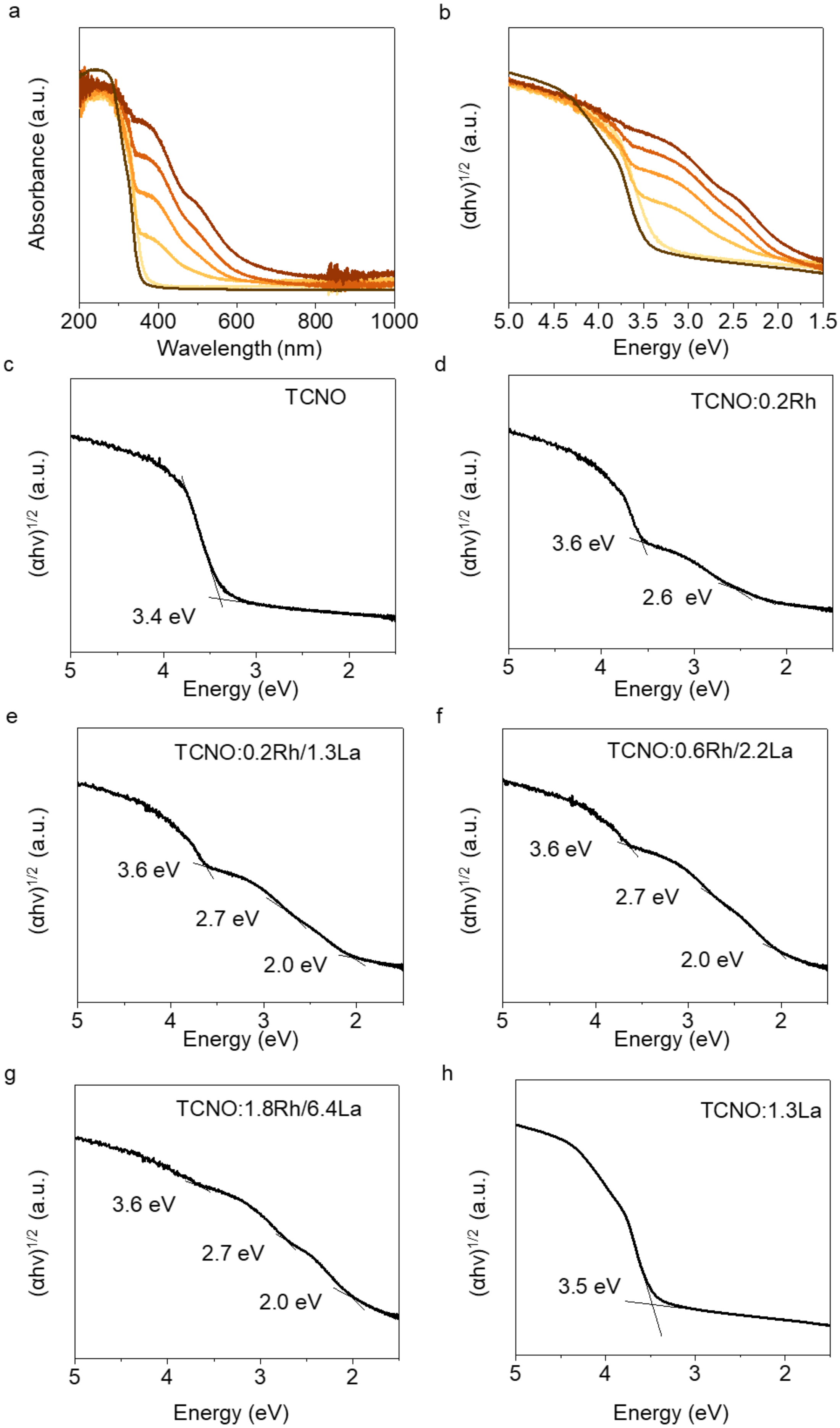

a
Absorbance (a.u.)
200 400 600 800 1000
Wavelength (nm)
b
(αhν)1/2 (a.u.)
5.0 4.5 4.0 3.5 3.0 2.5 2.0 1.5
Energy (eV)
c
TCNO
3.4 eV
d
TCNO:0.2Rh
3.6 eV
2.6 eV
e
TCNO:0.2Rh/1.3La
3.6 eV
2.7 eV
2.0 eV
f
TCNO:0.6Rh/2.2La
3.6 eV
2.7 eV
2.0 eV
g
TCNO:1.8Rh/6.4La
3.6 eV
2.7 eV
2.0 eV
h
TCNO:1.3La
3.5 eV
5 4 3 2
Energy (eV)

Supporting Figure 31. The absorbance edge determined in the Tauc plot converted from UV-Vis DRS spectra of undoped and doped TCNO. (a) UV-vis DRS spectra of exfoliated nanosheets. The colors, ranging from light to dark, represent the following samples in sequence: TCNO, TCNO:0.2Rh, TCNO:0.2Rh/1.3La, TCNO:0.6Rh/2.2La, TCNO:1.8Rh/6.4La. (b) TCNO. (c) TCNO:0.2Rh. (d) TCNO:0.2Rh/1.3La. (e) TCNO:0.6Rh/2.2La. (f) TCNO:1.8Rh/6.4La. (h) TCNO:1.3La

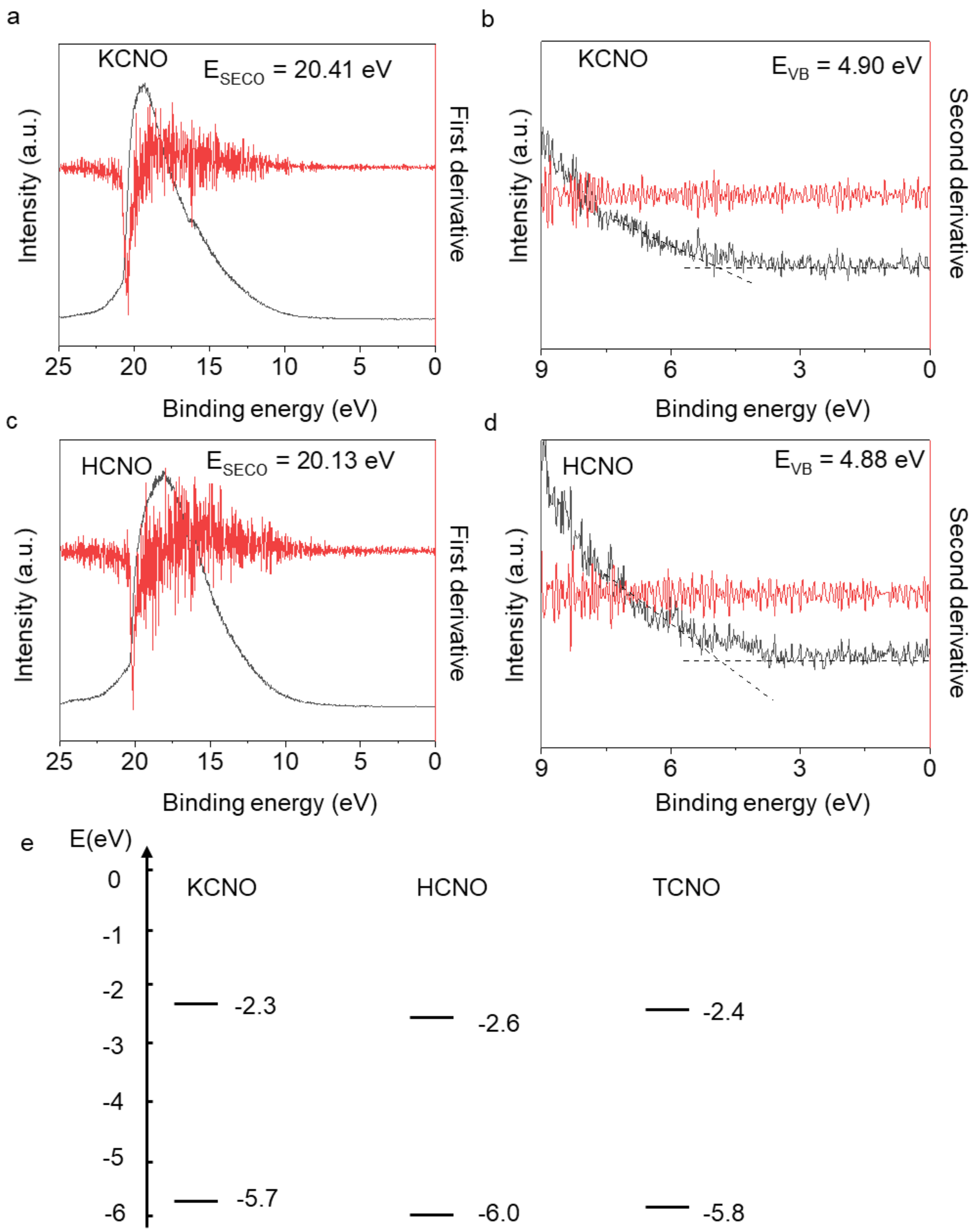

Supporting Figure 32. UPS and band edge position analysis of KCNO and HCNO. (a) and (b) KCNO. (c) and (d) HCNO. The secondary electron cut off is determined using first derivative method. The valence band maximum is determined using extrapolation method. (e) band edge position of KCNO, HCNO, and TCNO.

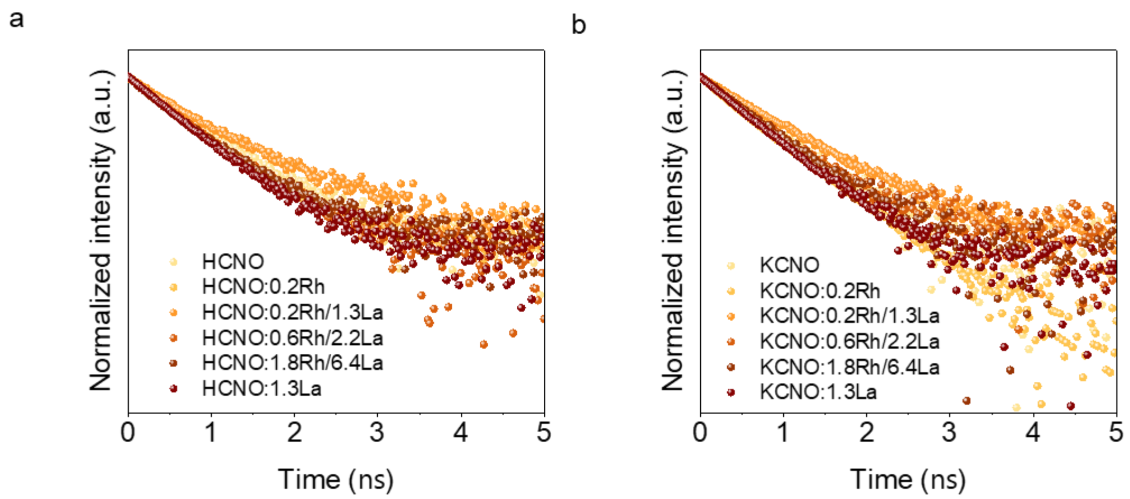

Supporting Figure 33. Normalized TRPL spectra of undoped and doped KCNO and HCNO monitored at 443 nm.

Supporting Table 1. Mechanism comparison between this work and selected literature.

Host Photocatalyst	Dopants	Mechanism for Enhanced Activity	Reference
$TBACa_2Nb_3O_{10}$	Rh/La	Trap mediated charge separation<br>• Rh acts as hole traps and mediated charge separation<br>• La acts as a structural stabilizer	This work
TiO_2	Rh/Nb	Charge compensation<br>Extended light absorption<br>Enhanced conductivity	6
$SrTiO_3$	Rh	Extended light absorption	7
$SrTiO_3$	La	Extended charge carrier lifetime	8
$SrTiO_3$	Rh/La	Charge compensation<br>Extended light absorption	9
$SrTiO_3$	Rh/La	Charge compensation<br>Enhanced reducing behavior	10
$SrTiO_3$	Rh/La	Charge compensation<br>Extended light absorption	11
$SrTiO_3$	Rh/La	Charge compensation<br>Enhanced charge carrier lifetime	12
$SrTiO_3$	Al/La	Charge compensation	13
$SrTiO_3$	Rh/Bi	Charge compensation	14
Sr_2TiO_4	Rh/La	Charge compensation<br>Extended light absorption<br>Enhanced charge separation and lifetime	15
$Ca_3Ti_2O_7$	Rh/Ln (Ln = La, Pr, Nd, Eu, Gd, Yb, and Y)	Charge compensation	16
$BaTiO_3$	Rh/La	Charge compensation	17
$BaZrO_3$	Rh/La	Charge compensation	17

Supporting Table 2a. Theoretical weight percentage of Ca, La, Nb, Rh and impurities in undoped and doped TCNO nanosheets.

	TCNO	TCNO: 0.2Rh	TCNO: 0.2Rh/1.3La	TCNO: 0.6Rh/2.2La	TCNO: 1.8Rh/6.4La
Ca (wt%) theoretical	22.34	22.32	21.98	21.29	18.98
La (wt%) theoretical	0.00	0.00	0.77	1.54	7.31
Nb (wt%) theoretical	77.66	76.82	76.40	76.32	69.65
Rh (wt%) theoretical	0.00	0.86	0.85	0.85	4.06
K (wt%) theoretical	0	0	0	0	0
Si (wt%) theoretical	0	0	0	0	0
Al (wt%) theoretical	0	0	0	0	0
Total (wt%)	100.0	100.0	100.00	100.0	100.0

Supporting Table 2b. Weight percentage of Ca, La, Nb, Rh and impurites in undoped and doped TCNO nanosheets determined by XRF. The analysis was performed using a factory-calibrated sensitivity library derived from reference standards, ensuring high measurement reliability. The method provides quantitative elemental compositions with good reproducibility, within approximately ±5% relative variation across repeated measurements.

	TCNO	TCNO: 0.2Rh	TCNO: 0.2Rh/1.3La	TCNO: 0.6Rh/2.2La	TCNO: 1.8Rh/6.4La
Ca (wt%) experiment	19.70	19.25	18.80	18.40	16.19
La (wt%) experiment	0.00	0.00	1.32	2.20	6.39
Nb (wt%) experiment	80.13	80.39	79.34	78.38	75.29
Rh (wt%) experiment	0.00	0.15	0.24	0.62	1.78
K (wt%) experiment	0.11	0.13	0.24	0.33	0.29
Si (wt%) experiment	0.05	0.05	0.03	0.05	0.04
Al (wt%) experiment	0.02	0.02	0.02	0.02	0.02
Total (wt%)	100.01	99.99	99.99	100.00	100.00

Note: The total weight percentage is not exactly 100% due to experimental uncertainties and inherent complexities of data collection and processing in XRF analysis.

Supporting Table 3. (Nb+Rh)/(Ca+La) atomic ratio in undoped and doped TCNO determined from XRF.

Sample	(Nb+Rh) : (Ca+La)
TCNO	3.5 : 2
TCNO:0.2Rh	3.6 : 2
TCNO:0.2Rh/1.3La	3.6 : 2
TCNO:0.6Rh/2.2La	3.6 : 2
TCNO:1.8Rh/6.4La	3.6 : 2

Supporting Table 4. BET surface area of representative bulk layered perovskites and exfoliated perovskites.

Sample	BET Surface Area ($m^2 g^{-1}$)
KCNO	0.47
KCNO:0.2Rh/1.3La	0.58
HCNO	0.92
HCNO:0.2Rh/1.3La	0.95
TCNO	35.17
TCNO:0.2Rh	30.71
TCNO:0.2Rh/1.3La	48.54
TCNO:0.6Rh/2.2La	47.87
TCNO:1.8Rh/6.4La	26.06
TCNO:1.3La	55.12

Supporting Table 5. Photocatalytic hydrogen evolution performance of the catalysts in this work evaluated by normalized units (mmol g^{-1} h^{-1}) and absolute units (μmol h^{-1}).

Photocatalyst	Hydrogen evolution activity mmol g^{-1} h^{-1}	Hydrogen evolution rate μmol h^{-1}
TCNO	2.4	12.3
TCNO:0.2Rh	11.8	62.7
TCNO:0.2Rh/1.3La	12.9	69.0
TCNO:0.6Rh/2.2La	5.0	26.1
TCNO:1.8Rh/6.4La	0.9	4.5
TCNO:1.3La	0.35	0.002

Supporting Table 6. Measurement parameters of wavelength-dependent apparent quantum efficiency (AQE).

Wavelength (nm)	Number of H_2 ($\times10^{18}$)	Power (mW)	Number of incident photons ($\times10^{19}$)	AQE (%)
300	3.97	3.52	1.90	41.75±1.73
330	2.71	4.11	2.45	22.09±1.22
365	3.09	4.50	2.97	20.80±0.95
405	3.48	7.31	5.35	12.99±0.88
437	2.19	6.95	5.50	7.98±1.20
650	Not detected	3.97	4.67	Not detected

Supporting Table 7. Photocatalytic activity of TCNO:0.2Rh/1.3La under one-hour illumination with a 420 nm long-pass filter, compared to that without the filter.

TCNO:0.2Rh/1.3La	Activity (μmol g^{-1} h^{-1})
With filter	10.95
No filter	12900.32

Supporting Table 8. Rh *K*-edge EXAFS fitting parameters of TCNO:0.2Rh/1.3La, TCNO:0.6Rh/2.2La, and TCNO:1.8Rh/6.4La.

Sample	Shell	Coordination Number	R (Å)	σ^2	R factor
TCNO:0.2Rh/1.3La	Rh-O	6.76 ± 0.44	2.030±0.005	0.0033	0.9%
TCNO:0.6Rh/2.2La	Rh-O	6.05 ± 0.48	2.047±0.006	0.0030	1.4%
TCNO:1.8Rh/6.4La	Rh-O	6.05 ± 0.54	2.040±0. 006	0.0030	1.7%

Supporting Table 9. Undoped and doped HCNO atomic ratio determined from XPS.

Sample	Rh^0(at%)	Rh^{3+}(at%)	Rh^0/Rh^{3+}	La^{3+}/Rh^{3+}
HCNO	0.00	0.00	-	-
HCNO:0.2Rh	32.17	67.83	0.47	0.00
HCNO:0.2Rh/1.3La	29.81	70.19	0.42	1.78
HCNO:0.6Rh/2.2La	23.62	76.38	0.31	3.60
HCNO:1.8Rh/6.4La	0.00	100.00	0.00	3.83

Supporting Table 10. Estimated weight percentage of Rh^0 and Rh^{3+} in undoped and doped TCNO.

Sample	Rh^0 (wt%)	Rh^{3+} (wt%)
TCNO	0.0	0.0
TCNO:0.2Rh	0.1	0.1
TCNO:0.2Rh/1.3La	0.1	0.2
TCNO:0.6Rh/2.2La	0.2	0.5
TCNO:1.8Rh/6.4La	0.0	1.8

Supporting Table 11. Energy of E_F, VBM, and CBM relative to vacuum level determined from UV-Vis and UPS.

Sample	E_F (eV)	VBM (eV)	CBM (eV)
TCNO	3.14	5.81	2.41
TCNO:0.2Rh	3.35	4.95	2.41
TCNO:0.2Rh/1.3La	3.18	4.98	2.98
TCNO:0.6Rh/2.2La	2.99	4.86	2.86
TCNO:1.8Rh/6.4La	3.18	4.99	2.99

Supporting Table 12. Bi-exponential fitting parameters of TRPL spectra.

Sample	A_1	τ_1 (ns)	A_2	τ_2 (ns)	$\tau_{average}$ (ns)
TCNO	0.33	0.56	0.67	1.03	0.93
TCNO:0.2Rh	0.30	0.45	0.70	0.93	0.84
TCNO:0.2Rh/1.3La	0.44	1.01	0.44	1.24	1.14
TCNO:0.6Rh/2.2La	0.41	0.79	0.41	0.97	0.89
TCNO:1.8Rh/6.4La	0.46	0.73	0.46	0.89	0.82
TCNO:1.3La	0.42	0.47	0.58	0.82	0.72
KCNO	0.51	0.48	0.49	0.79	0.67
KCNO:0.2Rh	0.37	0.37	0.63	0.67	0.6
KCNO:0.2Rh/1.3La	0.48	0.59	0.52	0.92	0.8
KCNO:0.6Rh/2.2La	0.42	0.41	0.58	0.72	0.63
KCNO:1.8Rh/6.4La	0.33	0.44	0.67	0.68	0.62
KCNO:1.3La	0.39	0.46	0.61	0.61	0.56
HCNO	0.41	0.46	0.59	0.79	0.69
HCNO:0.2Rh	0.45	0.42	0.55	0.68	0.59
HCNO:0.2Rh/1.3La	0.52	0.53	0.48	1.04	0.86
HCNO:0.6Rh/2.2La	0.41	0.38	0.59	0.74	0.65
HCNO:1.8Rh/6.4La	0.42	0.36	0.58	0.72	0.62
HCNO:1.3La	0.35	0.43	0.65	0.62	0.57
After reaction TCNO: 0.2Rh/1.3La	0.46	1.04	0.54	1.21	1.14

Supporting Table 13. Photocatalytic activity for hydrogen evolution of the catalysts in this work as compared to selected literature data.

Photocatalyst	Precious metal amount (wt%)	Light source	Reactant solution	Activity mmol g^{-1} h^{-1}	Reference
TCNO:0.2Rh	0.15	Xe lamp	10% methanol	11.8	This work
TCNO:0.2Rh/1.3La	0.24	Xe lamp	10% methanol	12.9	This work
Rh doped $TBACa_2Nb_3O_{10}$ nanosheets	0.86	UV	10% methanol	76.96	[3]
Pt/N/Nb doped $Ca_2NaNb_4O_{13}$- nanosheets	0.5	UV	20% methanol	9.73-19.46	[18]
$Pt/PA_2K_2Nb_6O_{17}$/(TBA, H) $Ca_2Nb_3O_{10}$	5.0	UV	20% methanol	31.2	[19]
Pt/Sillica pillared $HCa_2Nb_3O_{10}$	0.1	UV	10% methanol	8.1	[20]
Rh/N doped $CsCa_2Ta_3O_{10}$	0.15	Full arc	20% methanol	1.8	[21]

Supporting Note 1. XPS analysis

In the C 1s spectra, three adventitious carbon environments were observed. The most prominent component at the lowest binding energy was assigned to C-C environments and corrected to 284.8 eV, to calibrate the binding energy scale. The other two peaks at 286.3 eV and 288.4 eV were assigned to C-O and O-C=O environments, respectively.

The Ca 2p spectra showed a characteristic doublet with a 2p 3/2: 2p 1/2 peak area ratios of 2:1 and spin-orbit splitting of 3.5 eV.

In the Nb 3*d* spectra, the peaks were well fitted to two doublets with 3*d* 5/2: 3*d* 3/2 peak area ratios of 3:2 and spin orbit splitting of 2.7 eV. The doublets at lower binding energy were assigned to Nb^{4+}, and the doublets at higher binding energy was assigned to Nb^{5+}.[22] No obvious changes were observed on the atomic ratios between Nb^{5+} and Nb^{4+}.

The O 1s spectra displayed a reasonable fit to three components. The most intense peak at 530.4 eV was assigned to lattice O^{2-} in the bulk of HCNO.[23] The remaining peaks at 531.8 eV and 533.0 eV were assigned to the surface O-H group and adventitious carbon C-O respectively.

The La 3*d* spectra were fitted with a single component doublet resulting from spin-orbit splitting, with each spin-orbit component further split by multiplet splitting.

The Rh 3*d* spectra were fitted with doublets with 3*d* 5/2: 3*d* 3/2 peak area ratios of 3:2, and a spin-orbit splitting of 4.7 eV. Two components could fit the spectra reasonably. The doublet at around 307.7 eV for 3*d* 5/2 and 312.4 eV for 3*d* 3/2 was assigned to Rh^0. The doublet at around 309. 9 eV for 3d 5/2 and 314.5 eV for 3d 3/2 was assigned to Rh^{3+}.

Although the entire spectra of HCNO:1.8Rh/6.4La shifted 0.2 eV ~ 0.3 eV toward lower binding energy. However, considering the shift is around the instrumental resolution (~ 0.25 eV), it would not be attributed to chemical changes or shift of E_F.

Supporting Note 2. Correlation analysis of elemental and structural factors with catalytic activity.

Several other factors influencing the photocatalytic activity were examined:

All samples showed identical A/B site atomic ratios, excluding the influence of A/B site vacancies. Elemental weight percentages were semi-quantitatively determined by XRF (Supporting Table 2). In TCNO:0.2Rh, the measured Rh content was far less than the nominal value, indicating the difficulty of incorporating Rh into the TCNO lattice. With increasing La content, Rh incorporation improved, suggesting that La facilitates Rh doping. This low Rh incorporation raised the possibilities of B site vacancies. To evaluate this, the atomic ratios between B-site (Nb + Rh) and A-site (Ca + La) were analyzed based on XRF data (Supporting Table 3). The calculated value was larger than the ideal 3 : 2, seemingly implying a Nb-rich framework. However, this deviation was likely attributed to systematic error in the instrument and quantification method, as no supporting evidence of Nb-rich composition was found in XRD, XPS, and HAADF-STEM analysis. Besides, the ratios were consistent across all samples, indicating similar A-site and B-site occupancies between undoped and doped TCNO. Based on this, we exclude the influence of A/B site vacancies on the photocatalytic activity.

La^{3+} was found to be excessive. Beyond its role in charge compensation, this excess likely introduced recombination centers. The atomic ratio between Rh^{3+} (B site) and La^{3+} (A site) was quantified from XPS spectra (Supporting Table 9). The atomic ratio between Rh^{3+} and La^{3+} suggested that La^{3+} was excessive relative to the amount needed to compensate for the aliovalent doping of Rh^{3+}. This excess was pronounced in HCNO:0.6Rh/2.2La and HCNO:1.8Rh/6.4La, which partially accounted for their reduced charge carrier lifetime, increased recombination, and sharply declined activity.

Surface Rh^0 was not considered a dominant factor affecting photocatalytic activity. Although Rh^0 on the surface can serve as an electron reservoir due to its bigger work function (4.98 eV) than the nanosheets,[12] and thereby reduce SPV intensity, no clear correlation was observed between SPV signal and the estimated surface Rh^0 content. The SPV signals do not decrease with the increasing of estimated Rh^0 amount on the surface. Thus, we conclude that surface Rh^0 does not significantly influence the photocatalytic activity.

Supporting Note 3. Calculation method of wavelength-dependent apparent quantum efficiency (AQE).

A typical AQE calculation at 300 nm is shown below as an example:

The hydrogen amount analysed by GC was 6.59 μmol, corresponding to 3.97×10^{18} hydrogen molecules; During a period of 1 hour, the energy of the light irradiation: $W=P\times t$. With the bandpass filter of 300 nm, the power of incident light was measured to be P = 3.52 mW, therefore, the energy W= 0.00352 × 3600 = 12.67 J, which contains 1.90×10^{19} photons. Thus,

$$AQE(\%) = \frac{3.97 \times 10^{18} \times 2}{1.90 \times 10^{19}} \times 100\% = 41.75\%$$

Each AQE test was repeated three times, then the average value and standard deviation were calculated.

References

(1) Song, Y. J.; Iyi, N.; Hoshide, T.; Ozawa, T. C.; Ebina, Y.; Ma, R.; Miyamoto, N.; Sasaki, T. Accordion-Like Swelling of Layered Perovskite Crystals via Massive Permeation of Aqueous Solutions into 2D Oxide Galleries. *Chem. Commun.* **2015**, *51* (96), 17068–17071. DOI: 10.1039/c5cc05408g

(2) Sakaki, M.; Feng, Y. Q.; Kajiyoshi, K. Ultrasonic-Assisted Exfoliation of $Ca_2Nb_3O_{10}^-$ Nano-Sheets. *J. Solid State Chem.* **2019**, *277*, 253–259, DOI: 10.1016/j.jssc.2019.06.018

(3) Okamoto, Y.; Ida, S.; Hyodo, J.; Hagiwara, H.; Ishihara, T. Synthesis and Photocatalytic Activity of Rhodium-Doped Calcium Niobate Nanosheets for Hydrogen Production from a Water/Methanol System without Cocatalyst Loading. *J. Am. Chem. Soc.* **2011**, *133* (45), 18034–18037, DOI: 10.1021/ja207103j

(4) Compton, O. C.; Osterloh, F. E. Niobate Nanosheets as Catalysts for Photochemical Water Splitting into Hydrogen and Hydrogen Peroxide. *J. Phys. Chem. C* **2009**, *113* (1), 479–485, DOI: 10.1021/jp807839b

(5) Maheu, C.; Cardenas, L.; Puzenat, E.; Afanasiev, P.; Geantet, C. UPS and UV Spectroscopies Combined to Position the Energy Levels of TiO_2 Anatase and Rutile Nanopowders. *Phys Chem Chem Phys* **2018**, *20* (40), 25629–25637, DOI: 10.1039/c8cp04614j

(6) Huang, J.; Lv, T.; Huang, Q.; Deng, Z.; Chen, J.; Liu, Z.; Wang, G. Effect of Rh Valence State and Doping Concentration on the Structure and Photocatalytic H_2 Evolution in (Nb, Rh) Codoped TiO_2 Nanorods. *Nanoscale* **2020**, *12* (43), 22082–22090, DOI: 10.1039/D0NR05695B

(7) Iwashina, K.; Kudo, A. Rh-doped $SrTiO_3$ Photocatalyst Electrode Showing Cathodic Photocurrent for Water Splitting Under Visible-light Irradiation. *J. Am. Chem. Soc.* **2011**, *133* (34), 13272–13275, DOI: 10.1021/ja2050315

(8) Ichihara, F.; Sieland, F.; Pang, H.; Philo, D.; Duong, A.-T.; Chang, K.; Kako, T.; Bahnemann, D. W.; Ye, J. Photogenerated Charge Carriers Dynamics on La-and/or Cr-doped $SrTiO_3$ Nanoparticles Studied by Transient Absorption Spectroscopy. *J. Phys. Chem. C* **2020**, *124* (2), 1292–1302, DOI: 10.1021/acs.jpcc.9b09324

(9) Wang, Q.; Hisatomi, T.; Ma, S. S. K.; Li, Y.; Domen, K. Core/shell Structured La-and Rh-codoped $SrTiO_3$ as a Hydrogen Evolution Photocatalyst in Z-scheme Overall Water Splitting under Visible Light Irradiation. *Chem. Mater.* **2014**, *26* (14), 4144–4150, DOI: 10.1021/cm5011983

(10) Modak, B.; Ghosh, K.S. Exploring the Role of La Codoping beyond Charge Compensation for Enhanced Hydrogen Evolution by Rh–$SrTiO_3$. *J. Phys. Chem. B* **2015**, *119* (34), 11089–11098, DOI: 10.1021/acs.jpcb.5b02906

(11) Wei, Y.; Wan, J.; Wang, J.; Zhang, X.; Yu, R.; Yang, N.; Wang, D. Hollow Multishelled Structured $SrTiO_3$ with La/Rh Co-doping for Enhanced Photocatalytic Water Splitting under Visible Light. Small **2021**, 17 (22), 2005345, DOI: 10.1002/smll.202005345

(12) Moss, B.; Wang, Q.; Butler, K. T.; Grau-Crespo, R.; Selim, S.; Regoutz, A.; Hisatomi, T.; Godin, R.; Payne, D. J.; Kafizas, A; Domen, K.; Steier, L.; Durrant, J.R. Linking in situ Charge Accumulation to Electronic Structure in Doped $SrTiO_3$ Reveals Design Principles for Hydrogen-Evolving Photocatalysts. *Nat. Mater.* **2021**, *20* (4), 511–517, DOI: 10.5281/zenodo.4063942

(13) Qin, Y.; Fang, F.; Xie, Z.; Lin, H.; Zhang, K.; Yu, X.; Chang, K. La, Al-codoped $SrTiO_3$ as a Photocatalyst in Overall Water Splitting: Significant Surface Engineering Effects on Defect Engineering. *ACS Catal.* **2021**, *11* (18), 11429–11439, DOI: 10.1021/acscatal.1c02874

(14) Pan, Z.; Vequizo, J. J. M.; Yoshida, H.; Li, J.; Zheng, X.; Chu, C.; Wang, Q.; Cai, M.; Sun, S.; Katayama, K.; Yamakata, A.; Domen, K. Simultaneous Structural and Electronic Engineering on Bi-and Rh-co-doped $SrTiO_3$ for Promoting Photocatalytic Water Splitting. *Angew Chem Int Ed Engl* **2025**, *137* (2), e202414628, DOI: 10.1002/ange.202414628

(15) Sun, X.; Xu, X. Efficient Photocatalytic Hydrogen Production over La/Rh co-doped Ruddlesden-Popper Compound Sr_2TiO_4. *Appl. Catal., B* **2017**, *210*, 149–159, DOI: 10.1016/j.apcatb.2017.03.063

(16) Nishimoto, S.; Okazaki, Y.; Matsuda, M.; Miyake, M. Photocatalytic H_2 Evolution by Layered perovskite $Ca_3Ti_2O_7$ codoped with Rh and Ln (Ln= La, Pr, Nd, Eu, Gd, Yb, and Y) under Visible Light Irradiation. *J. Ceram. Soc. Jpn.* **2009**, *117* (1371), 1175–1179, DOI: 10.2109/jcersj2.117.1175

(17) Small, T. D.; Hamza, M. A.; Shen, Y.; Shearer, C. J.; Metha, G. F. Systematic Investigation of ABO_3 Perovskite Synthesis to Generalise a La and Rh co-doping Strategy for Visible Light Photocatalysis. *Nanoscale Adv., **2025**, 7, 4313-4324*, DOI: 10.1039/D5NA00338E

(18) Zhou, Y.; Wen, T.; Zhang, X.; Chang, B.; Kong, W.; Guo, Y.; Yang, B.; Wang, Y. A Multiple Structure-Design Strategy Toward Ultrathin Niobate Perovskite Nanosheets with Thickness-Dependent Photocatalytic Hydrogen-Evolution Performance. *Chem. - Asian J.* **2017**, *12* (20), 2727–2733, DOI: 10.1002/asia.201701001

(19) Zhou, H.; Sabio, E. M.; Townsend, T. K.; Fan, T.; Zhang, D.; Osterloh, F. E. Assembly of Core− Shell Structures for Photocatalytic Hydrogen Evolution from Aqueous Methanol. *Chem. Mater.* **2010**, *22* (11), 3362–3368, DOI: 10.1021/cm903839t

(20) Ebina, Y.; Tanaka, A.; Kondo, J. N.; Domen, K. Preparation of Silica Pillared $Ca_2Nb_3O_{10}$ and Its Photocatalytic Activity. *Chem. Mater.* **1996**, *8* (10), 2534–2538, DOI: 10.1021/cm960232q

(21) Ida, S.; Okamoto, Y.; Matsuka, M.; Hagiwara, H.; Ishihara, T. Preparation of Tantalum-Based Oxynitride Nanosheets by Exfoliation of a Layered Oxynitride, $CsCa_2Ta_3O_{10-x}N_y$, and Their Photocatalytic Activity. *J. Am. Chem. Soc.* **2012**, *134* (38), 15773–15782, DOI: 10.1021/ja3043678

(22) Koito, Y.; Rees, G. J.; Hanna, J. V.; Li, M. M. J.; Peng, Y. K.; Puchtler, T.; Taylor, R.; Wang, T.; Kobayashi, H.; Teixeira, I. F.; Khan, M.A.; Kreissl, H.T.; Tsang, S.C.E. Structure-Activity Correlations for Bronsted Acid, Lewis Acid, and Photocatalyzed Reactions of Exfoliated Crystalline Niobium Oxides. *Chemcatchem* **2017**, *9* (1), 144–154. DOI: 10.1002/cctc.201601131

(23) Zhang, W.; Uppuluri, R.; Mallouk, T. E.; Campbell, C. T. Silver Adsorption on Calcium Niobate(001) Nanosheets: Calorimetric Energies That Explain Sinter-Resistant Support. *J. Am. Chem. Soc.* **2020**, *142* (37), 15751–15763. DOI: 10.1021/jacs.0c05044

(24) Wang, J.; Zhao, J.; Osterloh, F. Photochemical Charge Transfer Observed in Nanoscale Hydrogen Evolving Photocatalysts Using Surface Photovoltage Spectroscopy. *Energy Environ. Sci.* **2015**, *8* (10), 2970–2976, DOI: 10.1039/C5EE01701G
